# Aryl Urea Based Scaffolds for Multitarget Drug Discovery in Anticancer Immunotherapies

**DOI:** 10.3390/ph14040337

**Published:** 2021-04-06

**Authors:** Celia Martín-Beltrán, Raquel Gil-Edo, Germán Hernández-Ribelles, Raül Agut, Pilar Marí-Mezquita, Miguel Carda, Eva Falomir

**Affiliations:** Departament de Química Inorgànica i Orgànica, Universitat Jaume I, E-12071 Castellón, Spain; beltranc@uji.es (C.M.-B.); ragil@uji.es (R.G.-E.); gribelle@uji.es (G.H.-R.); ragut@uji.es (R.A.); al107334@alumail.uji.es (P.M.-M.); mcarda@uji.es (M.C.)

**Keywords:** styryl aryl urea, phenethyl aryl urea, PD-L1, VEGFR-2, c-Myc, multitarget inhibitors, immunomodulation, angiogenesis

## Abstract

Twenty-one styryl and phenethyl aryl ureas have been synthetized and biologically evaluated as multitarget inhibitors of Vascular endothelial growth factor receptor-2 VEGFR-2 and programmed death-ligand-1 (PD-L1) proteins in order to overcome resistance phenomena offered by cancer. The antiproliferative activity of these molecules on several tumor cell lines (HT-29, MCF-7, HeLa and A549), on the endothelial cell line human microvascular endothelial cells (HMEC)-1 and on the non-tumor cell line human embryonic kidney cells (HEK)-293 has been determined. Some derivatives were evaluated for their antiangiogenic properties such as their ability to inhibit microvessel formation using HMEC-1 or their effect on VEGFR-2 in both cancer and endothelial cell lines. In addition, the immunomodulator action of a number of selected compounds was also studied on PD-L1 and c-Myc proteins. Compounds 16 and 23 (*Z*) and (*E*)-styryl *p*-bromophenyl urea, respectively, showed better results than sorafenib in down-regulation of VEGFR-2 and also improved the effect of the anti-PD-L1 compound BMS-8 on both targets, PD-L1 and c-Myc proteins.

## 1. Introduction

Cancer is synonymous with complex and multifactorial disease, however, it has been shown that at the cellular and molecular level a number of factors and hallmarks converge in all cancer cells regardless of the tissue or organ where they are placed [1,2]. Among these factors, the one that stands out and that has attracted the attention of many researchers in the last years is that related to cancer cells expressing some immune inhibitory proteins which cause immune cell dysfunction [3]. For this reason, immune evasion has become a major challenge for the development of successful cancer treatments [4].

One of the inhibitory proteins engaged in immune evasion is programmed death-ligand-1 (PD-L1) which binds to programmed death-1 (PD-1), which is expressed on immune T-cells, suppresses the action of this protein against cancer cells [5]. One of the specific proteins that regulate PD-L1 expression in cancer cells is c-Myc [6], a transcription factor that plays also a crucial role in the regulation of many other cancerous proteins [7]. Previous studies demonstrated that c-Myc promotes up regulation of PD-L1 [8].

Another important factor target that may influence the effectiveness of cancer immunotherapies is angiogenesis [9,10]. It has been demonstrated that normalization of tumor vasculature, by targeting pro-angiogenic tyrosine kinase receptor, Vascular endothelial growth factor-2 (VEGFR-2), reprogram the tumor microenvironment and enhance cancer immunotherapy [11].

**Figure 1:**
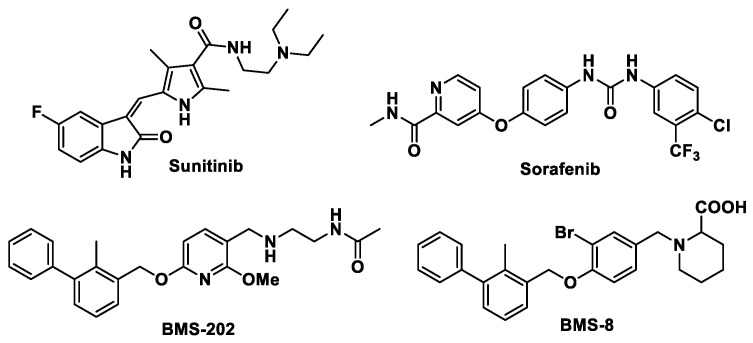
Structures of vascular endothelial growth factor-2 (VEGFR-2) inhibitors sunitinib and sorafenib and anti-programmed death-ligand-1 (PD-L1) agents BMS-202 and BMS-8.

Multikinase inhibitors, such as sunitinib or sorafenib (Figure 1), are capable to inhibit VEGFR-2 and have been approved by the FDA and the EMA for clinical use [12,13]. On the other hand, Brystol-Myers Squibb reported in 2015 the first non-peptidic small molecules able to interact with PD-L1. The mode of interaction of these compounds named BMS-202 and BMS-8 (Figure 1) was established in 2016 [14]. The identification of the binding site of these molecules in PD-L1 and their mechanism of action has opened the way for the design of new compounds able to disrupt PD-1/PD-L1 interaction [15].

Considering that the complexity of the tumor microenvironment also influences the immunosuppressive capacity of cancer cells, it seems obvious that monotargeted drug treatment may be insufficient for combating cancer. For this reason, multitarget drug therapies have emerged in recent years as one of the most promising cancer treatments [16,17]. 

Over the last five years we have been focused on the design, synthesis and biological evaluation of anticancer multitarget agents [18]. In this sense, our aim is to achieve potential anticancer drugs simultaneously targeting VEGFR-2, PD-L1 and c-Myc [19,20]. 

By means of docking studies on VEGFR-2 and PD-L1 proteins we have previously designed some scaffolds for potential multitarget inhibitors [20]. For this rational design we based on structures of VEGFR-2 inhibitor sorafenib and on PD-L1 inhibitor BMS-202. In the binding of sorafenib to kinase domain of VEGFR-2 urea functionality plays a key role by stablishing hydrogen bonding to glutamate and aspartate residues in the kinase binding pocket [21]. On the other hand, molecules such as BMS-202, developed by Brystol-Myers, inhibit the interaction between PD-1 and PD-L1 by binding to a PD-L1 hydrophobic groove formed by the amino acids Tyr56, Met115, Ile116, Ala121 and Tyr123 thus altering dimerization of PD-L1 protein. This mode of interaction achieves inhibition through a dual pathway: one is related with the filling by the inhibitor of the PD-1/PD-L1 interaction region which results in the blocking of this connection, and the other one is based on the modification of the PDL-1 dimer in which one of the proteins has the opposite configuration to that required for binding to PD-1. Consequently, the interaction between PD-1 and PD-L1 is disabled [14].

Both, the binding site in the kinase domain of VEGFR-2 and the binding site in PD-L1, possess a hydrophobic groove in which aromatic rings can be accommodated. For the design of compounds able to interact with both binding sites we carried out docking studies using Autodock 4.2 [22] on the kinase domain of VEGFR-2 and on the site identified in PD-L1 in order to find relatively simple structures able to interact with both sites. Several general structures mimicking the hydrogen bonds network shown by sorafenib were identified (Supplementary Materials, Figure S1). These structures are characterized by having a urea, carbamate or guanidinium moieties, which are essential to allow interaction with the kinase domain, to which aromatic rings are bound in order to interact with the hydrophobic grooves of both proteins.

**Figure 2:**
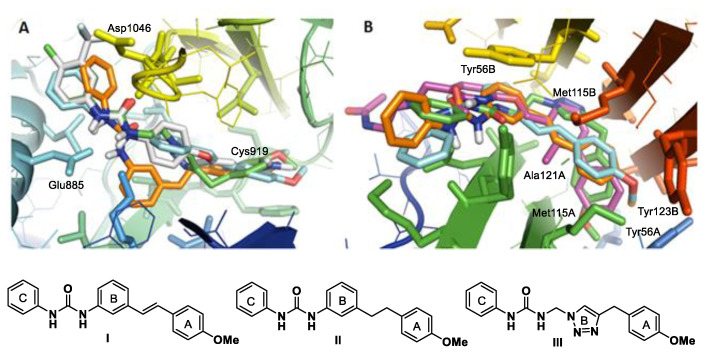
(**A**) Superposition of Compounds I (orange), II (light blue), III (green) and sorafenib (gray) in the kinase domain of VEGFR-2. (**B**). Superposition of Compounds I (orange), II (light blue), III (green) and BMS-202 (violet) at the PD-L1 binding site.

Based on these scaffolds, three families of aryl ureas derivatives were studied by docking (Figure 2) [20]. It can be appreciated in Figure 2A that Compounds I and II occupy the binding site in the kinase domain of VEGFR-2 in much the same way as sorafenib does [21]. These compounds establish hydrogen bonds between the OMe group and Cys919, between the two NH of the urea system and Glu885 and between the C = O of urea and NH backbone of Asp1046. All the aromatic rings interact with two hydrophobic zones present in the binding site. It can be seen in Figure 2B that Structures I and II also occupy the binding site of BMS-202 in PD-L1 protein. Aromatic rings, A, fit in a hydrophobic groove establishing interactions with Tyr56, Met115 from Chain A and Tyr123 from Chain B of PD-L1 dimer. Aromatic B rings establish hydrophobic interactions with Met115 from chain B and Ala121 from Chain A and aromatic rings, C, establish π-stacking interactions with Tyr56 from Chain B. The more polar urea system points towards the outer zone of the groove.

**Figure 3:**
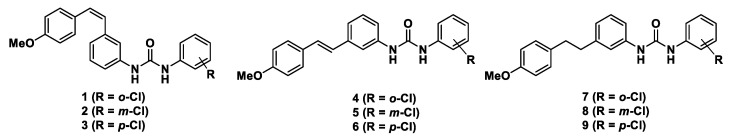
Potential multitarget inhibitors of VEFGR-2 and PD-L1.

In order to determine the validity of the results obtained in the in-silico design, we chose chlorophenyl Ureas 1–9 for a preliminary evaluation indicated in Figure 3 [20].

Our biological studies carried on compounds shown in Figure 3 validated aryl ureas as good scaffolds for designing multitarget inhibitors. For example, Compound 6, which bears a double bond of *E* configuration, showed the best antiangiogenic and immunomodulator properties and improved the effect of sorafenib as regards tube formation of endothelial cells and also improved the effect exerted by BMS-8 on both PD-L1 and c-Myc proteins [20].

**Scheme 1:**
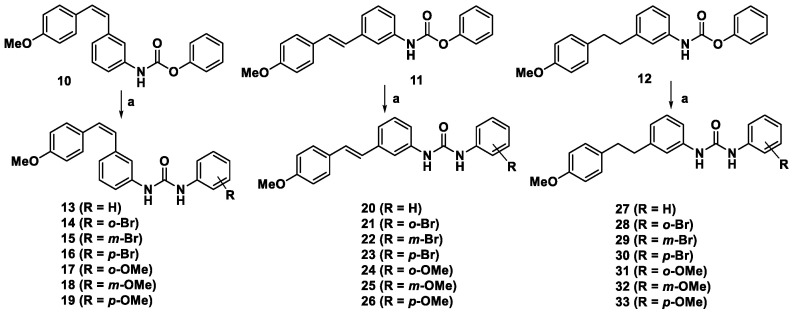
Synthetic route to the desired compounds. Reagents and conditions: (**a**) The appropriate aniline, Et_3_N, THF, 24–72 h, 40–50 °C.

The aim of the present work is to corroborate that the presence of the *E*-styryl unit in the aryl urea scaffold leads to the enhancement of the expected dual biological activity extending our previous study to new twenty-one derivatives showed in Scheme 1.

## 2. Results and Discussion

### 2.1. Synthesis of Aryl Urea Derivatives

Phenylcarbamates 10–12, synthesized as previously described [20], were transformed in the desired Ureas 13–33 by reaction with a set of anilines (see Scheme 1).

### 2.2. Biological Evaluation

#### 2.2.1. Cell Proliferation Inhibition

The ability of Ureas 13–33 to inhibit cell proliferation was established by means of their IC_50_ values towards the human tumor cell lines HT-29 (colon adenocarcinoma), MCF-7 (breast adenocarcinoma), HeLa (epithelioid cervix carcinoma) and A549 (pulmonary adenocarcinoma), as well as towards the endothelial cell line HMEC-1 (human microvascular endothelial cells) and the non-tumor cell line HEK-293 (human embryonic kidney cells). The IC_50_ values for Ureas 13–33 are presented in Table 1 along with IC_50_ values for the reference compounds sorafenib and BMS-8.

The synthetic ureas showed antiproliferative activity in the low micromolar range in all tested cell lines, comparable to that shown by reference compounds sorafenib and BMS-8. The (E)-styryl aryl ureas are more active than Z derivatives or saturated ones with IC_50_ values at submicromolar level. The most active derivatives are 22 (m-bromophenyl urea), 23 (p-bromophenyl urea) and 26 (p-methoxyphenyl urea). It is worth mentioning that the results for bromophenyl ureas are similar to the ones reported for chlorophenyl Derivatives 4–6 (see Figure 3). In this case, 5 (m-chlorophenyl urea) and 6 (p-chlorophenyl urea) exhibited IC_50_ values ranging from 2 to 11 μM and from 0.4 to 1.8 μM, respectively.

This study demonstrates that there is a correlation between the scaffold of the derivatives and IC_50_ values. Thus, (E)-styryl aryl ureas exhibit the lowest IC_50_ values, followed by phenethyl aryl ureas and finally (Z)-styryl aryl ureas. It seems that in this particular item (cell proliferation inhibition) the folded structure of (Z)-derivatives could be detrimental compared to linear conformation adopted by the (E)-derivatives and the greater conformational freedom of phenethyl derivatives. Some correlation of IC_50_ values and the position of the substituent on the aryl urea unit can also be observed, for instance for (E)-styryl bromophenyl Ureas 21–23 in which para-substitution increases inhibition of cell proliferation as regards meta or ortho substituted ones.

#### 2.2.2. Effect on Cellular VEGFR-2 in HT-29 and HMEC-1

To assess the anti-angiogenic properties of these derivatives we decided to study their effect on VEGFR-2, one of the targets that we chose for the designing of these scaffolds.

We performed the study on both a HT-29 cancer cell line and a human microvessel endothelial cell line (HMEC-1). HT-29 cell line was chosen by us due to the experience gained by our group in this type of cells. We also made a selection of eight derivatives based on both their IC_50_ values in HT-29 and on their structure in order to establish structure-activity relationships. The selected compounds were (Z)-styryl ureas 16, 17 and 19, (E)-styryl ureas 23, 25 and 26 and phenethyl ureas 32 and 33. The effect of these selected derivatives on VEGFR-2, on HT-29 tumor cell line and on human microvessel endothelial cells (HMEC) was determined by flow cytometry technique. Both membrane and total VEGFR-2 were relatively determined using dimethyl sulfoxide (DMSO) treated cells as a negative control and sorafenib as a positive one. For these assays, cells were incubated for 24 h in the presence of the corresponding compounds at 10 µM concentration. Table 2 shows the effects achieved by the selected compounds on membrane and total VEGFR-2, in both cell lines and to DMSO as control.

All the derivatives were more effective in inhibiting membrane VEGFR-2 than the total one in both HT-29 and HMEC-1. The most active compounds were (Z) and (E)-styryl-p-bromo aryl Ureas 16 and 23, respectively, showing an inhibition of more than 50% of both membrane and total VEGFR-2 and improving the effect exerted by sorafenib. 

#### 2.2.3. Effect on Microtube Formation on Endothelial Cells

The capacity to inhibit the formation of new vasculature network formed by HMEC-1 was evaluated on Compounds 16, 23 and 33, which showed as the most active VEGFR-2 inhibitors. Table 3 shows the minimum concentration at which these compounds are active and begin to inhibit the microtube formation.

**Figure 4:**
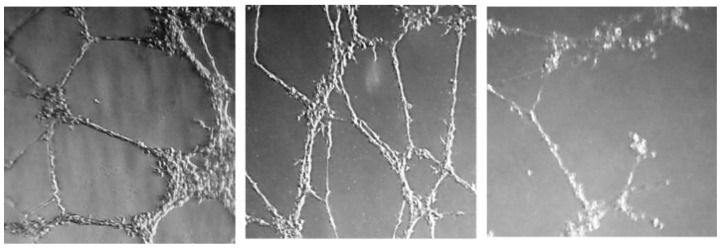
Effect of **23** at 0.25, 0.5 and 1 µM concentrations (from left to right).

Pictures for the inhibition of neovascularization achieved by Compound 23, at different concentrations are displayed in Figure 4.

A comparison of the minimum active concentration values to IC_50_ values for HMEC-1 cell line (see Table 1) shows that there is a correlation between antiproliferative activity and tube formation inhibition capacity, as compounds with lower IC_50_ values exhibit microtube inhibition activity at lower concentrations. Moreover, it is observed that two of the tested compounds (E) styryl-p-bromo aryl urea 23 and phenethyl p-methoxy aryl urea 33 are more active than sunitinib and sorafenib in this particular issue.

#### 2.2.4. Effect on PD-L1 and on c-Myc Proteins

To assess immunomodulator properties of our derivatives, we decided to study their effect on PD-L1 and on c-Myc, a transcription factor that regulates PD-L1 production.

We performed the study on two cancer cell lines, HT-29 and A-549, to check the versatility of their potential activity. The study included the same selected derivatives as for VEGFR-2: 16, 17 and 19, (E)-styryl ureas 23, 25 and 26 and phenethyl ureas 32 and 33.

We evaluated the effect of the selected compounds on PD-L1 and on c-Myc proteins by flow cytometry after 24 h treatment at 10 μM concentration of compounds. Both, PD-L1 and c-Myc were relatively determined using DMSO treated cells as a negative control and BMS-8 as a positive one. Table 4 shows the percentage of total proteins detected for each compound referred to control (DMSO) on both cell lines.

From data shown in Table 4 it can be deduced that Compounds 16, 23 and 33 are the most active inhibiting PD-L1 in both cancer cell lines, displaying a stronger effect than that exerted by BMS-8. On the other hand, again (E)-styryl p-bromophenyl Ureas 16 and 23 are the most active ones inhibiting both PD-L1 and c-Myc proteins.

#### 2.2.5. Cell Proliferation Evaluation in Co-Cultures

**Figure 5:**
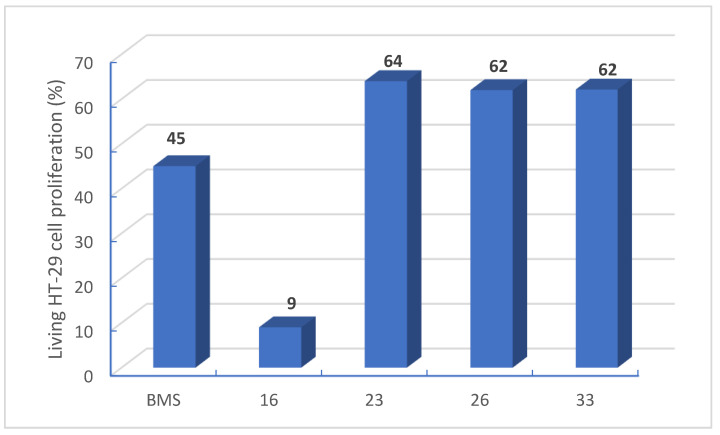
Percentage of HT-29 cell proliferation (%) in presence of Jurkat T cells. Data are representative of five experiments.

We also studied the effect of Compounds 16, 23, 26 and 33 on tumor cell proliferation in the presence of PD-1 expressing Jurkat T-cells. For this assay, first interferon γ stimulated HT-29 cells were treated for 24 h with the selected compounds at 10 µM in presence of Jurkat T cells. Then, living cells were counted using trypan blue and a Neubauer chamber and by flow cytometry. Figure 5 shows the inhibition of tumor cell proliferation exhibited by the selected compounds due to the presence of Jurkat T cells.

Data provided in Figure 5 show that (Z)-styryl p-bromophenyl 16 is the most active compound exhibiting an inhibition of HT-29 cell proliferation of almost 90% when co-cultured with Jurkat T-cells, thus improving the effect exerted by BMS-8. This was a non-expected result because both 16 and 23 exhibited similar action on all the studied targets (PD-L1, c-Myc and also VEGFR-2) and the antiproliferative action of 16 in mono cultures of HT-29 was almost ten times lower than the one exerted by 23. Surprisingly, antiproliferative activity of Compound 16 was largely enhanced when the assay was performed in HT-29 co cultured with Jurkat T cells.

**Figure 6:**
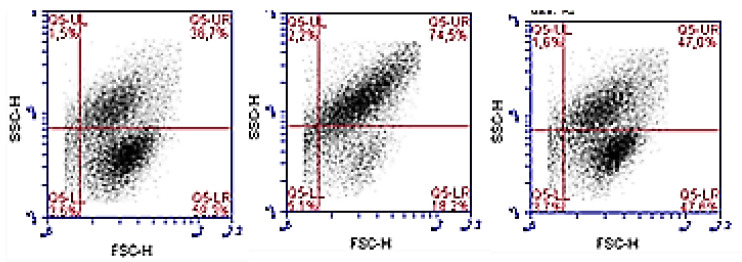
Jurkat T cell population distribution (%) co-cultured with HT-29 in the presence of dimethyl sulfoxide (DMSO), Compound 16 and BMS-8 from left to right. Data are representative of five experiments.

We also determine Jurkat T cell proliferation in those co-cultures from the corresponding media by flow cytometry. Figure 6 shows T cell population distribution by size (FSC-H, X axis) and granularity (SCC-H, Y axis). All samples provided similar population distribution except by those treated with Compound 16, in which an enhancement of size and complexity is observed.

**Figure 7:**
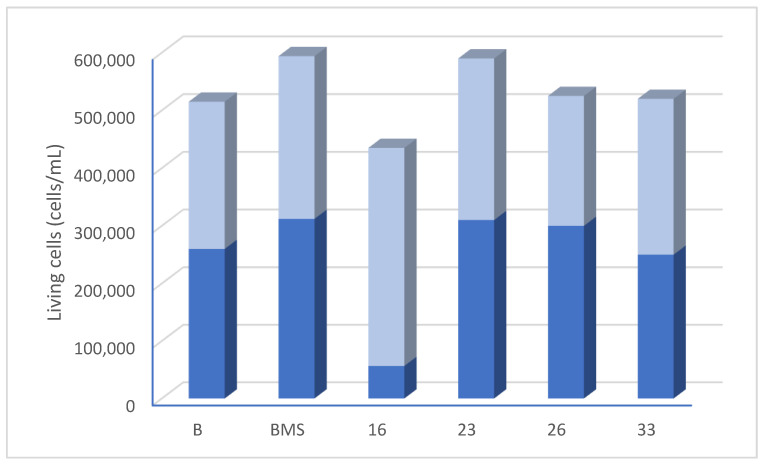
Percentage of Jurkat T cell proliferation and population distribution (%) co-cultured with HT-29. Data are representative of five experiments.

Data provided in Figure 7 show the density of living Jurkat T cells in the treated co-cultures with HT-29. The graphics include cell distribution population by size and complexity in each case.

Data provided in Figure 6 and Figure 7 show that none of the derivatives exerted an important effect neither on the Jurkat T cell proliferation nor on T cell population distribution. (Z)-styryl p-bromophenyl 16 is the one than, again, behaves different from the rest of tested compounds. In this case, 16 hardly affect proliferation of T cell but this compound enhances T cell morphological changes causing an increase on their size and complexity. These unexpected and singular results for Compound 16 let us conclude that its mechanism of action is different from the rest of tested compounds pointing to an activation of T cell activity against cancer cells. More studies will be further carried out in order to better establish the mode of action of Compound 16.

## 3. Materials and Methods

### 3.1. Chemistry

#### 3.1.1. General Procedures

^1^H and ^13^C NMR spectra were measured at 25 °C. The signals of the deuterated solvent (DMSO_d6_) were taken as the reference. Multiplicity assignments of ^13^C signals were made by means of the DEPT pulse sequence. Complete signal assignments in ^1^H and ^13^C NMR spectra were made with the aid of 2D homo—and heteronuclear pulse sequences (COSY, HSQC, HMBC). High resolution mass spectra were recorded using electrospray ionization–mass spectrometry (ESI–MS). IR data were measured with oily films on NaCl plates (oils) and are given only for relevant functional groups (C = O, NH). Experiments which required an inert atmosphere were carried out under dry N_2_ in flame-dried glassware. Commercially available reagents were used as received.

#### 3.1.2. Experimental Procedure for the Synthesis of Compounds **13–33**

A solution of the corresponding aniline (0.5 mmol) in dry THF (4 mL/mmol) was treated with Et_3_N (5.4 mmol) under inert atmosphere. After stirring the mixture for 5 min, the corresponding previously prepared carbamate (0.5 mmol) was added dropwise as a solution in tetrahydrofuran THF (10 mL/mmol). The resulting mixture was then stirred in the dark at 40–50 °C for 24–72 h (TLC monitoring). After this time, CH_2_Cl_2_ (15 mL) and aqueous HCl 1M were added to the reaction mixture, which was then extracted twice with CH_2_Cl_2_ (10 mL). The organic layer was washed with brine and then dried on anhydrous Na_2_SO_4_. Removal of volatiles under reduced pressure afforded an oily residue which was subjected to column chromatography on silica-gel (hexane-EtOAc 4:1 as eluent) to afford the desired products. Detailed analytical data are given in the Supplementary Material.

### 3.2. Biological Studies

#### 3.2.1. Cell Culture

Cell culture media were purchased from Gibco (Grand Island, NY, USA). Fetal bovine serum (FBS) was obtained from Harlan-Seralab (Belton, UK). Supplements and other chemicals not listed in this section were obtained from Sigma Chemical Co. (St. Louis, MO, USA). Plastics for cell culture were supplied by Thermo Scientific BioLite. For tube formation assay, an IBIDI μ-slide angiogenesis (IBIDI, Martinsried, Germany) were used. All tested compounds were dissolved in DMSO at a concentration of 10 mM and stored at −20 °C until use.

HT-29, MCF-7, HeLa, A549 and HEK-293 cell lines were maintained in Dulbecco’s modified Eagle’s medium (DMEM) containing glucose (1 g/L), glutamine (2 mM), penicillin (50 μg/mL), streptomycin (50 μg/mL), and amphotericin B (1.25 μg/mL), supplemented with 10% FBS. HMEC-1 cell line was maintained in Dulbecco’s modified Eagle’s medium (DMEM)/low glucose containing glutamine (2 mM), penicillin (50 μg/mL), streptomycin (50 μg/mL), and amphotericin B (1.25 μg/mL), supplemented with 10% FBS. For the development of tube formation assays in Matrigel, HMEC-1 cells were cultured in EGM-2MV Medium supplemented with EGM-2MV SingleQuots.

#### 3.2.2. Cell Proliferation Assay

In 96-well plates, 3 × 10^3^ (HeLa, A549, HMEC-1, HEK-293) or 5 × 10^3^ (HT-29, MCF-7) cells per well were incubated with serial dilutions of the tested compounds in a total volume of 100 μL of their respective growth media. The 3-(4,5-dimethylthiazol-2-yl)-2,5-diphenyltetrazolium bromide (MTT; Sigma Chemical Co.) dye reduction assay in 96-well microplates was used. After 2 days of incubation (37 °C, 5% CO_2_ in a humid atmosphere), 10 μL of MTT (5 mg/mL in phosphate-buffered saline, PBS) was added to each well, and the plate was incubated for a further 3 h (37 °C). After that, the supernatant was discarded and replaced by 100 µL of DMSO to dissolve formazan crystals. The absorbance was then read at 540 nm by spectrophotometry. For all concentrations of compound, cell viability was expressed as the percentage of the ratio between the mean absorbance of treated cells and the mean absorbance of untreated cells. Three independent experiments were performed, and the IC_50_ values (i.e., concentration half inhibiting cell proliferation) were graphically determined using GraphPad Prism 4 software.

#### 3.2.3. VEGFR-2 Quantification by Flow Cytometry

VEGFR-2 was determined quantifying Alexa Fluor^®^ 647 Mouse Anti-Human CD309 (VEGFR-2) by means of flow cytometry. To detect membrane VEGFR-2, cells were incubated with compounds for 24 h and then they were collected, fixed and stained with Alexa Fluor^®^ 647 Mouse Anti-Human CD309 (VEGFR-2). For the detection of total VEGFR-2 (membrane and cytosolic), cells were incubated with compounds for 24 h, then lysates were obtained and stained with Alexa Fluor^®^ 647 Mouse Anti-Human CD309 (VEGFR-2).

#### 3.2.4. Tube Formation Inhibition Assay

Wells of an IBIDI μ-slide angiogenesis (IBIDI, Martinsried, Germany) were coated with 12 μL of Matrigel^®^ (10 mg/mL, BD Biosciences) at 4 °C. After gelatinization at 37 °C for 30 min, HMEC-1 cells were seeded at 2 × 10^4^ cells/well in 35 μL of culture medium on top of the Matrigel and were incubated 30 min at 37 °C while are attached. Then, compounds were added dissolved in 35 μL of culture medium and after 20 h of incubation at 37 °C, tube destruction was evaluated.

#### 3.2.5. PD-L1 and c-Myc Quantification by Flow Cytometry and Enzyme Linked Immunosorbent Assay (ELISA)

For the detection of total PD-L1 and c-Myc, cancer cells were seeded (3 × 10^5^ cells/well) in 6-well plates and were incubated with the corresponding compounds for 24 h. Then they were collected, fixed, treated with 0.5% Triton^TM^ X-100 and stained with Alexa Fluor^®^ 647 Rabbit monoclonal Anti-PD-L1 (ab215251) and FITC Rabbit monoclonal anti-c-Myc (ab223913). 

We also checked results performing ELISA tests. For ELISA tests, lysates were collected, protein quantification was carried out by Bradford test and, then, PD-L1 and c-Myc were quantified using Human PD-L1 ELISA Kit 28-8 (ab214565) and c-Myc (Total) Human ELISA Kit (KHO2041), respectively, according to the manufacturer’s instructions.

#### 3.2.6. Cell Proliferation Evaluation in Co-Cultures

In 12-well plates, 10^5^ HT-29 cells per well were seeded and incubated for 24 h with cell culture medium supplemented with IFN-γ (10 ng/mL; human, Invitrogen^®^) then medium was change by one containing 4 × 10^5^ Jurkat T cells per well and 10 μM of the corresponding compound or DMSO for the control. After 24 h of incubation, supernatants were collected to determine Jurkat T cell living cells and, on the other hand, HT-29 cells were collected with trypsin. Both types of suspension cells were counted with the Neubauer chamber and trypan blue and also by flow cytometry.

## Figures and Tables

**Table 1:** IC_50_ values (μM) for sorafenib, BMS-8 and Derivatives 13–33.

Comp.	HT-29	MCF-7	HeLa	A549	HMEC-1	HEK-293
**Sorafenib**	17 ± 4	14 ± 4	6.1 ± 0.4	27 ± 2	34 ± 3	5.0 ± 0.7
**BMS-8**	19 ± 2	20 ± 3	>100	6 ± 1	-	60 ± 10
**13**	24 ± 5	30 ± 7	32 ± 8	34 ± 2	40 ± 5	16 ± 3
**14**	17 ± 2	25 ± 10	26 ± 3	32 ± 14	14 ± 2	25 ± 3
**15**	25 ± 2	28 ± 5	30 ± 7	38 ± 6	24 ± 4	23 ± 2
**16**	17 ± 1	24 ± 7	12 ± 4	12 ± 2	27 ± 8	10 ± 2
**17**	15 ± 1	15 ± 1	15 ± 2	18 ± 1	10 ± 1	27 ± 5
**18**	17 ± 4	19 ± 2	24 ± 5	23 ± 8	4.3 ± 0.3	16 ± 3
**19**	14 ± 3	8 ± 3	5 ± 2	12 ± 2	11 ± 1	10 ± 1
**20**	2.5 ± 0.8	2.3 ± 0.9	42 ± 8	1.6 ± 0.3	4 ± 1	15 ± 2
**21**	1.6 ± 0.2	1.6 ± 0.3	1.7 ± 0.4	3 ± 1	1.9 ± 0.4	2.0 ± 0.2
**22**	0.5 ± 0.2	0.6 ± 0.3	1.5 ± 0.3	4 ± 3	1.1± 0.2	0.7 ± 0.3
**23**	0.4 ± 0.3	0.3 ± 0.2	3.1 ± 0.1	3 ± 0.4	1.8 ± 0.3	0.7 ± 0.3
**24**	11 ± 4	3.6 ± 0.6	9 ± 4	11 ± 5	7.2± 0.6	3.5 ± 0.8
**25**	1.3 ± 0.5	1.1 ± 0.2	7.0 ± 0.5	2.2 ± 0.7	4.3 ± 0.4	4 ± 2
**26**	0.2 ± 0.2	0.7 ± 0.3	4 ± 1	13 ± 3	1.9 ± 0.6	1.4 ± 0.6
**27**	9.6 ± 0.6	12.8 ± 0.9	10 ± 2	12 ± 5	9 ± 4	12.5 ± 0.2
**28**	7.9 ± 0.5	5 ± 4	4 ± 3	7 ± 4	5 ± 3	1.7 ± 0.2
**29**	11 ± 2	10 ± 4	10.7 ± 0.5	13 ± 3	3.8 ± 0.3	7.2 ± 0.4
**30**	4.9 ± 0.8	4 ± 2	3.7 ± 0.3	11 ± 5	1.3 ± 0.1	1.3 ± 0.5
**31**	57 ± 6	10 ± 5	24 ± 2	24 ± 5	>100	13.8 ± 0.8
**32**	22 ± 2	25 ± 4	27 ± 1	18 ± 3	7.1 ± 0.8	9.7 ± 0.8
**33**	4.4 ± 0.8	1.5 ± 0.6	1.4 ± 0.5	8 ± 3	0.9 ± 0.03	0.9 ± 0.07

IC_50_ values are expressed as the compound concentration that inhibits the cell growth by 50%. Data are the average (± standard deviation (SD)) of the three experiments.

**Table 2:** Effect on VEGFR-2 expression in HT-29 and human microvascular endothelial cells (HMEC) cells.

	HT-29		HMEC	
Comp.	Membrane VEGFR-2 (%)	Total VEGFR-2 (%)	Membrane VEGFR-2 (%)	Total VEGFR-2 (%)
**Sorafenib**	92 ± 3	85 ± 5	46 ± 8	64 ± 4
**16**	39 ± 9	55 ± 10	45 ± 8	59 ± 5
**17**	47 ± 16	98 ± 12	99 ± 7	97 ± 9
**19**	88 ± 14	97 ± 7	98 ± 8	99 ± 16
**23**	36 ± 9	39 ± 8	39 ± 5	74 ± 8
**25**	99 ± 11	94 ± 7	59 ± 8	96 ± 9
**26**	68 ± 9	95 ± 9	65 ± 9	83 ± 7
**32**	76 ± 5	93 ± 10	69 ± 8	98 ± 13
**33**	68 ± 13	100 ± 10	66 ± 5	93 ± 8

Data are the average (±SD) of three experiments.

**Table 3:** Inhibition effect of tube formation.

Comp.	Minimum Active Conc. (µM)
**Sunitinib**	3
**Sorafenib**	10
**16**	10
**23**	1
**33**	6

Data are the average of three experiments.

**Table 4:** PD-L1 and c-Myc protein relative expression on HT-29 and A-549 cell lines.

	HT-29		A-549	
Comp.	PD-L1 (%)	c-Myc (%)	PD-L1 (%)	c-Myc (%)
**BMS-8**	62 ± 3	99 ± 4	68 ± 5	60 ± 7
**16**	17 ± 5	41 ± 10	23 ± 8	29 ± 5
**17**	60 ± 12	58 ± 13	79 ± 9	45± 9
**19**	36 ± 9	47 ± 8	98 ± 10	79 ± 10
**23**	18 ± 5	49 ± 8	16 ± 6	34 ± 4
**25**	69 ± 11	74 ± 11	59 ± 8	68 ± 9
**26**	33 ± 10	57 ± 9	50 ± 3	63 ± 7
**32**	55 ± 11	69 ± 7	59 ± 9	77 ± 15
**33**	38 ± 13	70 ± 10	42 ± 11	83 ± 12

Data are the average (±SD) of three experiments.

## Data Availability

Not applicable.

## Supplementary Materials

The following are available online at https://www.mdpi.com/article/10.3390/ph14040337/s1, Figure S1: Scaffolds able to interact with the kinase domain of VEGFR-2 and PD-L1; S-3/S-7: Analytical NMR spectra and S-8/S-41: Graphical NMR data.
