# Supplementary material for: Aryl Urea Based Scaffolds for Multitarget Drug Discovery in Anticancer Immunotherapies
Source: Pharmaceuticals (Basel). 2021 Apr 6;14(4):337. doi: 10.3390/ph14040337 (PMC8067507; doi:10.3390/ph14040337)
Supplement: Supplementary file 1 [file pharmaceuticals-14-00337-s001.pdf]

Aryl Urea based Scaffolds for Multitarget Drug Discovery in Anticancer Immunotherapies

Celia Martín-Beltrán [1], Raquel Gil-Edo [1], German Hernández-Ribelles [1], Raül Agut [1], Pilar Marín-Mezquita [1], Miguel Carda [1,*] and Eva Falomir [1,*]

[1] Depart. de Q. Inorgánica y Orgánica, Univ. Jaume I, E-12071 Castellón, Spain.
* Correspondence: efalomir@uji.es; mcarda@uji.es

Supporting Information

Contents:

S-2: ***Figure S1***

S-3/S-7: ***Analytical NMR spectra***

S-8/S-41: ***Graphical NMR data***

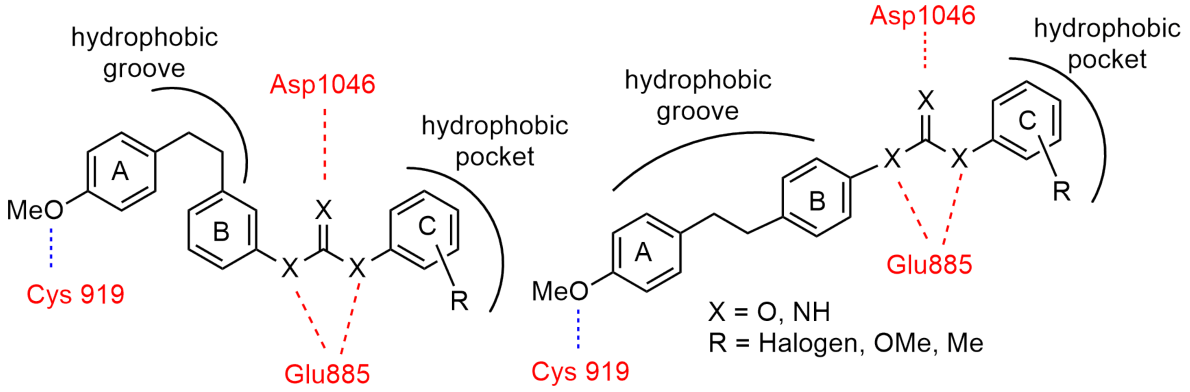

Figure S1. Scaffolds able to interact with the kinase domain of VEGFR-2 and PD-L1.

The hydrogen bonds that these compounds can establish with VEGFR-2 are indicated in red. The regions of the molecules that could interact with the hydrophobic pockets of both binding sites are also indicated.

Analytical NMR spectra

(*Z*)-1-(2-bromophenyl)-3-(3-(4-methoxystyryl)phenyl)urea (14): Yield 41 %; ^{1}H NMR (400 MHz, DMSO$_{d6}$) δ 9.38 (s, 1H); 8.10 (s, 1H); 8.00 (dd, *J* = 8.0, 4.0 Hz, 1H); 7.60 (d, *J* = 4.0 Hz, 1H); 7.30 (d, *J* = 4.0 Hz, 1H); 7.20 (dd, *J* = 8.0, 4.0 Hz, 2H); 7.20-7.17 (m, 3H); 6.94 (dd, *J* = 12.0, 4.0 Hz, 1H); 6.85 (d, *J* = 12.0 Hz, 1H); 6.82 (d, *J* = 8.0 Hz, 2H); 6.55 (d, *J* = 12.0, 8.0 Hz, 2H); 3.70 (s, 3H); ^{13}C NMR (100 MHz, DMSO$_{d6}$) δ 158.4 (C); 152.0 (C); 139.6 (C); 138.0 (C); 137.0 (C); 132.4 (x2 CH); 129.9 (CH); 129,7 (C); 128.9 (C); 128.2 (C); 128.0 (C); 124.0 (C); 122.2 (CH); 118.0 (CH); 113.7 (CH); 113.0 (CH_3) 55.1; IR ν_{max} (cm^{-1}) 3332 (NH), 1555 (CO); HR ESMS *m/z* 423.0675 [M+Na]$^{+}$. Calc. for $C_{22}H_{19}O_2N_2Br$, 423.0715.

(*Z*)-1-(3-bromophenyl)-3-(3-(4-methoxystyryl)phenyl)urea (15): Yield 62 %, white solid, m. p. 174 °C; ^{1}H NMR (400 MHz, DMSO$_{d6}$) δ 8.78 (s, 1H); 8.65 (s, 1H); 7.83 (s, 1H); 7.39-7.15 (m, 8H); 6.82 (t, *J* = 8.0 Hz, 3H); 6.51 (d, *J* = 12.0 Hz, 1H); 6.50 (d, *J* = 12.0 Hz, 1H); 3.75 (s, 3H); ^{13}C NMR (100 MHz, DMSO$_{d6}$) δ 158.6 (C); 152.4 (C); 140.2 (C); 139.9 (C); 138.6 (C); 131.2 (CHx2); 130.0 (CH); 129.6 (CH); 128.4 (CH); 127.7 (CHx2); 124.9 (CH); 123.2 (CH); 122.8 (CH); 120.1 (CHx2); 118.2 (CH); 117.3 (CH); 113.5 (CHx2); 55.2 (CH_3); IR ν_{max} (cm^{-1}) 3287 (NH), 1649 (CO); HR ESMS *m/z* 423.0711 [M+H]$^{+}$. Calc. for $C_{22}H_{19}O_2N_2Br$, 423.0715.

(*Z*)-1-(4-bromophenyl)-3-(3-(4-methoxystyryl)phenyl)urea (16): Yield 58 %, white solid, m. p. 173 °C; ^{1}H NMR (400 MHz, DMSO$_{d6}$) δ 8.75 (s, 1H); 8.63 (s, 1H); 7.41(d, *J* = 8.1 Hz, 2H); 7.45-7-39 (m, 3H); 7.28-7.17 (m, 4H); 6.84-6.80 (m, 3H); 6.51 (dd, *J* = 12.0, 8.0 Hz, 2H); 3.72 (s, 3H); ^{13}C NMR (100 MHz, DMSO$_{d6}$) δ 158.5 (C); 152.5 (C); 139.8 (C); 138.6 (C); 137.6 (C); 131.5 (CH x 2); 129.9 (CH); 129.6 (CH); 128.2 (CH); 127.5 (CHx2); 126.0 (CH); 122.5(CH); 122.2 (CH); 120.1 (CHx2); 118.1 (CH); 117.0 (CH); 113.8 (CHx2); 55.0 (CH_3); IR ν_{max} (cm^{-1}) 3291 (NH), 1633 (CO); HR ESMS *m/z* 423.0708 [M+H]$^{+}$. Calc. for $C_{22}H_{19}O_2N_2Br$, 423.0711.

(*Z*)-1-(2-methoxyphenyl)-3-(3-(4-methoxystyryl)phenyl)urea (17): Yield 31 %; ^{1}H NMR (400 MHz, $DMSO_{d6}$) δ 9.22 (s, 1H); 8.18 (s, 1H); 8.10 (d, *J* = 8.0 Hz, 1H); 7.29 (d, *J* = 8.0 Hz, 1H); 7.20 (s, 1H); 7.18-7.10 (m, 3H); 7.0 (d, *J* = 4.0 Hz, 1H); 6.94-6.86 (m, 3H); 6.83 (d *J* = 4.0 Hz, 1H); 6.51 (dd, *J* = 12.0, 4.0 Hz, 1H); 3.4 (s, 3H); 3.3 (s, 3H); ^{13}C NMR (100 MHz, $DMSO_{d6}$) δ 158.4 (C); 152.3 (C); 147.0 (C); 140.0 (C); 138.1 (C); 129.9 (2xCH); 129.6 (CH); 128.8 (C); 128.8 (CH); 128.2 (CH), 121.8 (CH); 121.7 (CH); 120.5 (CH); 118.2 (CH); 117.7 (CH); 116.7 (CH); 110.7 (CH); 55.7 (CH_3); 55.0 (CH_3); IR ν_{max} (cm^{-1}) 3302 (NH), 1595 (CO); HR ESMS *m/z* 375.1867 [M+H]$^+$. Calc. for $C_{23}H_{22}O_3N_2$, 375.1865.

(*Z*)-1-(3-methoxyphenyl)-3-(3-(4-methoxystyryl)phenyl)urea (18): Yield 61 %; ^{1}H NMR (400 MHz, $DMSO_{d6}$) δ 8.61 (s, 1H); 8.57 (s, 1H); 7.38 (d, *J* = 12.0 Hz, 1H); 7.30 (s, 1H); 7.20-7.14 (m, 5H); 6.90 (d, *J* = 12.0 Hz, 1H); 6.85-6.81 (m, 3H); 6.51 (dd, *J* = 12.0, 8.0 Hz, 3H); 3.7 (s, 3H); 3.3 (s, 3H); ^{13}C NMR (100 MHz, $DMSO_{d6}$) δ 158.4 (C); 152.3 (C); 147.0 (C); 140.0 (C); 138.1 (C); 129.6 (2xCH); 129.4 (CH); 129.2 (C); 128.5 (CH); 128.0 (CH), 121.7 (CH); 117.8 (CH); 116.7 (CH); 113.4 (CH); 110.2 (CH); 107.0 (CH), 103.7 (CH), 54.7 (CH_3); 54.6 (CH_3); IR ν_{max} (cm^{-1}) 3307 (NH), 1595 (CO); HR ESMS *m/z* 375.1887 [M+H]$^+$. Calc. for $C_{23}H_{22}O_3N_2$, 375.1866.

(*Z*)-1-(4-methoxyphenyl)-3-(3-(4-methoxystyryl)phenyl)urea (19): Yield 66 %; ^{1}H NMR (400 MHz, $DMSO_{d6}$) δ 8.60 (s, 1H); 8.51 (s, 1H); 7.65 (s, 1H); 7.56 (d, *J* = 8.0 Hz, 2H); 7.37 (d, *J* = 8.0 Hz, 2H); 7.30-7.20 (m, 3H); 7.15 (d, *J* = 12.0 Hz, 2H); 6.95 (d, *J* = 8.0 Hz, 2H); 6.87 (d, *J* = 12.0 Hz, 2H); 3.78 (s, 3H); 3.72 (s, 3H); ^{13}C NMR (100 MHz, $DMSO_{d6}$) δ 159.0 (C); 154.5 (C); 152.7 (C); 140.2 (C); 137.8 (C); 132.7 (CH); 129.6 (CH); 129.2 (C); 129.0 (CH); 128.0 (2xCH); 127.8 (CH); 126.3 (CH); 120.0 (2xCH); 119.6 (CH); 117,1 (CH); 115.8 (CH); 114.1 (CH); 114.0 (2xCH); 55.1 (2xCH_3); IR ν_{max} (cm^{-1}) 3310 (NH), 1575 (CO); HR ESMS *m/z* 375.1867 [M+H]$^+$. Calc. for $C_{23}H_{22}O_3N_2$, 375.1856.

(*E*)-1-(2-bromophenyl)-3-(3-(4-methoxystyryl)phenyl)urea (21): Yield 13 %, white solid, m. p. 230-232 °C; ^{1}H NMR (400 MHz, $DMSO_{d6}$) δ 9.48 (s, 1H); 8.15 (s, 1H); 8.09 (d, *J* = 8.0 Hz, 1H); 7.68 (s, 1H); 7.62 (d, *J* = 8.0 Hz, 1H); 7.56 (d, *J* = 8.0 Hz, 2H); 7.37-7.21 (m, 4H); 7.10 (d, *J* = 8.0 Hz, 2H); 6.98 (d, *J* = 8.03 Hz, 1H); 6.95 (d, *J* = 12.0 Hz, 2H), 3.78 (s, 3H); ^{13}C NMR (100 MHz, $DMSO_{d6}$) δ 159.0 (C); 152.2 (C); 138.8 (C); 138.0 (C); 132,4 (C); 129.5 (C); 129.1 (C); 129.1 (CH); 128.1 (CH); 128,0 (x 2) (CH); 127.8 (CH); 126.1 (CH); 124,0 (CH); 121.9 (CH); 122,2 (CH); 120.1 (CH); 117.3 (CH); 115.9 (CH); 114.1 (x 2) (CH); 113,0, (CH); 55.0 (CH_3); IR ν_{max} (cm^{-1}) 3692 (NH), 1425 (CO); HR ESMS *m/z* 423.0677 [M+H]$^+$. Calc. for $C_{22}H_{19}O_2N_2Br$, 423.0713.

(*E*)-1-(3-bromophenyl)-3-(3-(4-methoxystyryl)phenyl)urea (22): Yield 66 %; ^{1}H NMR (400 MHz, DMSO$_{d6}$) δ 8.88 (s, 1H); 8.74 (s, 1H); 7.89 (s, 1H); 7.68 (s, 1H); 7.56 (d, *J* = 8.0 Hz, 2H); 7.32-7.29 (m, 6H); 7.10 (d, *J* = 12.0 Hz, 2H); 6.94 (d, *J* = 12.0 Hz, 2H); 3.78 (s, 3H); ^{13}C NMR (100 MHz, DMSO$_{d6}$) δ 159.0 (C); 152.4 (C) ; 141.4 (C); 139.7 (C); 137.9 (C); 130.6 (CH); 129.5 (CH); 129.0 (CH); 128.0 (CH); 127.8 (CH); 126.2 (CH); 124.9 (CH); 121.7 (CH); 120.5 (CH); 117.5 (CH); 117.0 (CH); 116.1 (CH); 114.1 (CH); 55.1(CH_3); IR ν_{max} (cm^{-1}) 3544 (NH), 1425 (CO); HR ESMS *m/z* 423.0867 [M+H]$^{+}$. Calc. for $C_{22}H_{19}O_2N_2Br$, 423.0687.

(*E*)-1-(4-bromophenyl)-3-(3-(4-methoxystyryl)phenyl)urea (23): Yield 82 %; ^{1}H NMR (400 MHz, DMSO$_{d6}$) δ 8.88 (s, 1H); 8.75 (s, 1H); 7.66 (s, 1H); 7.56 (d, *J* = 8.0 Hz, 2H); 7.45 (s, 4H); 7.25-7.21 (m, 3H); 7.12 (dd, *J* = 16.0, 12 Hz, 2H); 6.95 (d, *J* = 12.0 Hz, 2H); 3.78 (s, 3H); ^{13}C NMR (100 MHz, DMSO$_{d6}$) δ 159.0 (C); 152.4 (C); 139.8 (C); 139.1 (C); 137.8 (C); 131.4 (CH); 129.5 (CH); 129.0 (CH); 128.0 (CH); 127.8 (CH); 126.2 (CH); 120.1 (CH); 120.0 (CH); 117.4 (CH); 116.1 (CH); 114.1 (CH); 113.1 (CH); 55.1 (CH_3); IR ν_{max} (cm^{-1}) 3695(NH), 1431 (CO); HR ESMS *m/z* 423.0698 [M+H]$^{+}$. Calc. for $C_{22}H_{19}O_2N_2Br$, 423.0656.

(*E*)-1-(2-methoxyphenyl)-3-(3-(4-methoxystyryl)phenyl)urea (24): Yield 31 %; ^{1}H NMR (400 MHz, DMSO$_{d6}$) δ 9.33 (s, 1H); 8.25 (s, 1H); 8.15 (d, *J* = 4.0 Hz, 1H); 7.68 (s, 1H); 7.56 (d, *J* = 8.0 Hz, 2H); 7.27 (dd, *J* = 8.0, 4.0 Hz, 2H); 7.25 (d, *J* = 8.0 Hz, 1H); 7.19 (d, *J* = 8.0 Hz, 2H); 7.18-6.90 (m, 5H); 3.89 (s, 3H); 3.78 (s, 3H); ^{13}C NMR (100 MHz, DMSO$_{d6}$) δ 159.0 (C); 152.4 (C); 147.6 (C); 140.0 (C); 137.9 (C); 129.6 (CH); 129.0 (CH); 128.7 (C); 128.0 (CH); 127.8 (x2 CH); 126.3 (CH); 121.8 (CH); 120.5 (CH); 119.7 (CH); 118.5 (CH); 117.0 (CH); 115.7 (CH); 114.5 (CH); 110.7 (CH); 55.7 (CH_3); 55.1 (CH_3); IR ν_{max} (cm^{-1}) 3313 (NH), 1593 (CO); HR ESMS *m/z* 375.1856 [M+H]$^{+}$. Calc. for $C_{23}H_{22}O_3N_2$, 375.1861.

(*E*)-1-(3-methoxyphenyl)-3-(3-(4-methoxystyryl)phenyl)urea (25): Yield 45 %; ^{1}H NMR (400 MHz, DMSO$_{d6}$) δ 8.73 (s, 1H); 8.69 (s, 1H); 7.65 (s, 1H); 7.56 (d, *J* = 8.0 Hz, 2H); 7.28-7.20 (m, 7H); 6.95 (d, *J* = 8.0 Hz, 3H); 6.56 (d, *J* = 12.0 Hz, 1H); 3.78 (s, 3H); 3.74 (s, 3H); ^{13}C NMR (100 MHz, DMSO$_{d6}$) δ (C) 158.4 (C); 152.3 (C); 147.0 (C); 140.0 (C); 138.1 (C); 129.6 (2xCH); 129.4 (CH); 129.2 (C); 128.5 (CH); 128.0 (CH); 121.7 (CH); 117.8 (CH); 116.7 (CH); 113.4 (CH); 110.2 (CH); 107.0 (CH); 103.7 (CH); 54.7 (CH_3); 54.6 (CH_3); IR ν_{max} (cm^{-1}) 3307 (NH), 1595 (CO); HR ESMS *m/z* 375.1887 [M+H]$^{+}$. Calc. for $C_{23}H_{22}O_3N_2$, 375.1866.

(*E*)-1-(4-methoxyphenyl)-3-(3-(4-methoxystyryl)phenyl)urea (26): Yield 70 %; ^{1}H NMR (400 MHz, DMSO$_{d6}$) δ 8.55 (s, 1H); 8.45 (s, 1H); 8.23 (s, 1H); 7.99 (d, *J* = 8.0 Hz, 2H); 7.90 (d, *J* = 12.0 Hz, 2H); 7.83 (d, *J* = 12.0 Hz, 1H); 7.67 (t, *J* = 8.0 Hz, 2H); 7.62 (d, *J* = 8.0 Hz, 1H); 7.53 (s, 1H); 7.39 (d, *J* = 8.0 Hz, 2H); 7.32 (d, *J* = 12.0 Hz, 2H); 4.27 (s, 3H); 4.32 (s, 3H); ^{13}C NMR (100 MHz, DMSO$_{d6}$) δ (C) 158,4 (C); 154.4 (C); 152.6 (C); 140.0 (C); 137.7 (C); 132.6 (2xCH); 129.9 (CH); 129.6 (C); 128.9 (CH); 128.7 (CH); 128.3 (CH); 121.6 (CH); 120.0 (2xCH); 117.9 (CH); 116,9 (CH); 114,0 (CH), 113,7 (CH), 55,1 (2xCH_3); IR ν_{max} (cm^{-1}) 3320 (NH), 1565 (CO); HR ESMS *m/z* 375.1869 $[M+H]^+$. Calc. for $C_{23}H_{22}O_3N_2$, 375.1846.

1-(2-bromophenyl)-3-(3-(4-methoxyphenethyl)phenyl)urea (28): Yield 12 %; ^{1}H NMR (400 MHz, DMSO$_{d6}$) δ 9.38 (s, 1H), 8.09 (d, *J* =8.0 Hz 1H); 8.06 (s, 1H); 7.61 (d, *J* = 8.0 Hz, 2H); 7.33 (s, 1H); 7.29 (t, *J* = 8.9 Hz, 2H); 7.28 (d, *J* = 8.0 Hz, 1H); 7.18 (t, *J* = 8.0 Hz, 1H); 7.13 (d, *J* = 8.0 Hz, 2H); 7.02 (t, *J* = 8.0 Hz, 1H); 6.84 (d, *J* = 12.0 Hz, 1H); 6.82 (d, *J* = 8.0 Hz, 2 H); 3.70 (s, 3H); 2.81 (s, 4H); ^{13}C NMR (100 MHz, DMSO$_{d6}$) 157.4 (C); 152.3 (C); 142.2 (C); 141.4 (C); 139.3 (C); 133.3 (C); 130.7 (CH); 129.2 (2xCH); 128.6 (CH); 124.3 (CH); 122.3 (CH); 121.7 (C); 120.3 (CH); 118.3 (CH); 117.0 (CH); 116.0 (CH); 113.6 (2xCH); 54.9 (CH_3); 37.5 (CH_2); 36.1 (CH_2).IR ν_{max} (cm^{-1}) 3301 (NH), 1645 (CO); HR ESMS *m/z* 425.08778$[M+H]^+$. Calc. for $C_{22}H_{21}N_2O_2$, 425.0866.

1-(3-bromophenyl)-3-(3-(4-methoxyphenethyl)phenyl)urea (29): Yield 14 %; ^{1}H NMR (400 MHz, DMSO$_{d6}$) δ 8.84 (s, 1H); 8.66 (s, H); 7.87 (t, *J* = 2.1 Hz, 1H); 7.4 (t, *J* = 2.1 Hz, 1H); 7.25-7.12 (m, 7H); 6.84-6.82 (m, 3H); 3.71 (s, 3H); 2.81 (s, 4H); ^{13}C NMR (100 MHz, DMSO$_{d6}$) δ 157.4 (C); 152.3 (C); 142.2 (C); 141.4 (C); 139.3 (C); 133.3 (C); 130.7 (CH); 129.2 (2xCH); 128.6 (CH); 124.3 (CH); 122.3 (CH); 121.7 (C); 120.3 (CH); 118.3 (CH); 117.0 (CH); 116.0 (CH); 113.6 (2xCH); 54.9 (CH_3); 37,5 (CH_2); 36,1 (CH_2).; IR ν_{max} (cm^{-1}) 3304 (NH), 1633 (CO); HR ESMS *m/z* 425.08473 $[M+Na]^+$. Calc. for $C_{22}H_{21}BrN_2O_2$, 425.0865.

1-(4-bromophenyl)-3-(3-(4-methoxyphenethyl)phenyl)urea (30): Yield 27 %; ^{1}H NMR (400 MHz, DMSO$_{d6}$) δ 8.80 (s, 1H); 8.70 (s, 1H); 7.42 (s, 4H); 7.31 (s, 1H); 7.30 (t, *J* = 7.5 Hz, 1H); 7.25 (m, 3H); 7.18 (d, *J* = 7.5 Hz, 3H); 3.71 (s, 3H); 2.81 (s, 4H); ^{13}C NMR (100 MHz, DMSO$_{d6}$) δ 157.4 (C); 152.4 (C); 142.2 (C); 139.4 (C); 139.2 (C); 133.3 (C); 131.5 (CH); 129.3 (2xCH); 128.6 (CH); 122.2 (CH); 122.0 (CH); 120.1 (CH); 118.3 (CH); 115.9 (CH); 113.6 (2xCH); 113.1 (CH); 54.9 (CH_3); 37.4 (CH_2); 36.1 (CH_2).; IR ν_{max} (cm^{-1}) 3290 (NH), 1638 (CO); HR ESMS *m/z* 425.0877 $[M-H]^-$. Calc. for $C_{22}H_{21}BrN_2O_2$, 425.0865.

1-(2-methoxyphenyl)-3-(3-(4-methoxyphenethyl)phenyl)urea (31): Yield 83 %; ^{1}H NMR (400 MHz, $DMSO_{d6}$) δ 9.24 (s, 1H); 8.20 (s, 1H); 8.13 (d, *J* = 8.0 Hz, 1H); 7.33 (s, 1H); 7.26 (d, *J* = 8.10Hz, 1H); 7.17-7.13 (m, 3H); 7.02-6.82 (m, 4H); 3.88 (s, 3H); 3.71 (s, 3H); 2.81 (s, 4H);^{13}C NMR (100 MHz, $DMSO_{d6}$) δ 157.6 (C); 152.6 (C); 147.9 (C); 142.4 (C); 139.8 (C); 133.5 (C); 129.5 (2xCH); 128.9 (CH); 128.8 (C); 122.2 (CH); 122.1 (CH); 120.7 (CH); 118.5 (CH); 118.1 (CH); 115.8 (CH); 113.8 (2xCH); 110.9 (CH); 55.9 (CH_3); 55.1 (CH_3); 37.6 (CH_2); 36.2 (CH_2); IR ν_{max} (cm^{-1}) 3301 (NH), 1645 (CO); HR ESMS *m/z* 377.1867 $[M+H]^+$. Calc. for $C_{23}H_{24}O_3N_2$, 377.1865.

1-(3-methoxyphenyl)-3-(3-(4-methoxyphenethyl)phenyl)urea (32): Yield 69 %; ^{1}H NMR (400 MHz, $DMSO_{d6}$) δ 8.70 (s, 1H); 8.56 (s, 1H); 7.33 (s, 1H); 7.25 (dd, *J* = 8.0, 4.0 Hz, 1H); 7.19-7.13 (m, 5H); 6.93 (d, *J* = 8.0 Hz, 1H); 6.83 (d, *J* = 8.0 Hz, 3H); 6.55 (d, *J* = 8.0 Hz, 1H); 3.73 (s, 3H); 3.71 (s, 3H); 2.80 (s, 4H); ^{13}C NMR (100 MHz, $DMSO_{d6}$) δ 159.7 (C); 157.4 (C); 152.4 (C); 142.2 (C); 141.0 (C); 139.5 (C); 133.4 (C); 129.5 (CH); 129.3 (2xCH); 128.6 (CH); 122.0 (CH); 118.2 (CH); 115.8 (CH); 113.6 (2xCH); 110.5 (CH); 107.2 (CH); 103.9 (CH); 54.9 (2xCH_3); 37.5 (CH_2); 36.1 (CH_2); IR ν_{max} (cm^{-1}) 3304 (NH), 1633 (CO); HR ESMS *m/z* 377.1866 $[M+H]^+$. Calc. for $C_{23}H_{24}N_2O_3$, 377.1865.

1-(4-methoxyphenyl)-3-(3-(4-methoxyphenethyl)phenyl)urea (33): Yield 98 %; ^{1}H NMR (400 MHz, $DMSO_{d6}$) δ 8.5 (s, 1H); 8.4 (s, H); 7.3 (t, *J* = 8.2 Hz, 3H); 7.2 (d, *J* = 8.2 Hz, 1H); 7.1 (t, *J* = 8.3 Hz, 3H); 6.8 (m, 5H); 3.7 (s, 3H); 2.8 (s, 4H); ^{13}C NMR (100 MHz, $DMSO_{d6}$) δ 157.4 (C); 154.4 (C); 152.7 (C); 142.1 (C); 139.8 (C); 133.4 (C); 132.7 (C); 129.2 (2xCH); 128.5 (CH); 121.8 (CH); 119.9 (2xCH); 118.0 (CH); 115.7 (CH); 113.9 (2xCH); 113.6 (2xCH); 55.1 (CH_3); 54.9 (CH_3); 37.5 (CH_2); 36.1 (CH_2); IR ν_{max} (cm^{-1}) 3290 (NH), 1638 (CO); HR ESMS *m/z* 377.1868 $[M-H]^-$. Calc. for $C_{23}H_{24}N_2O_3$, 377.1865.

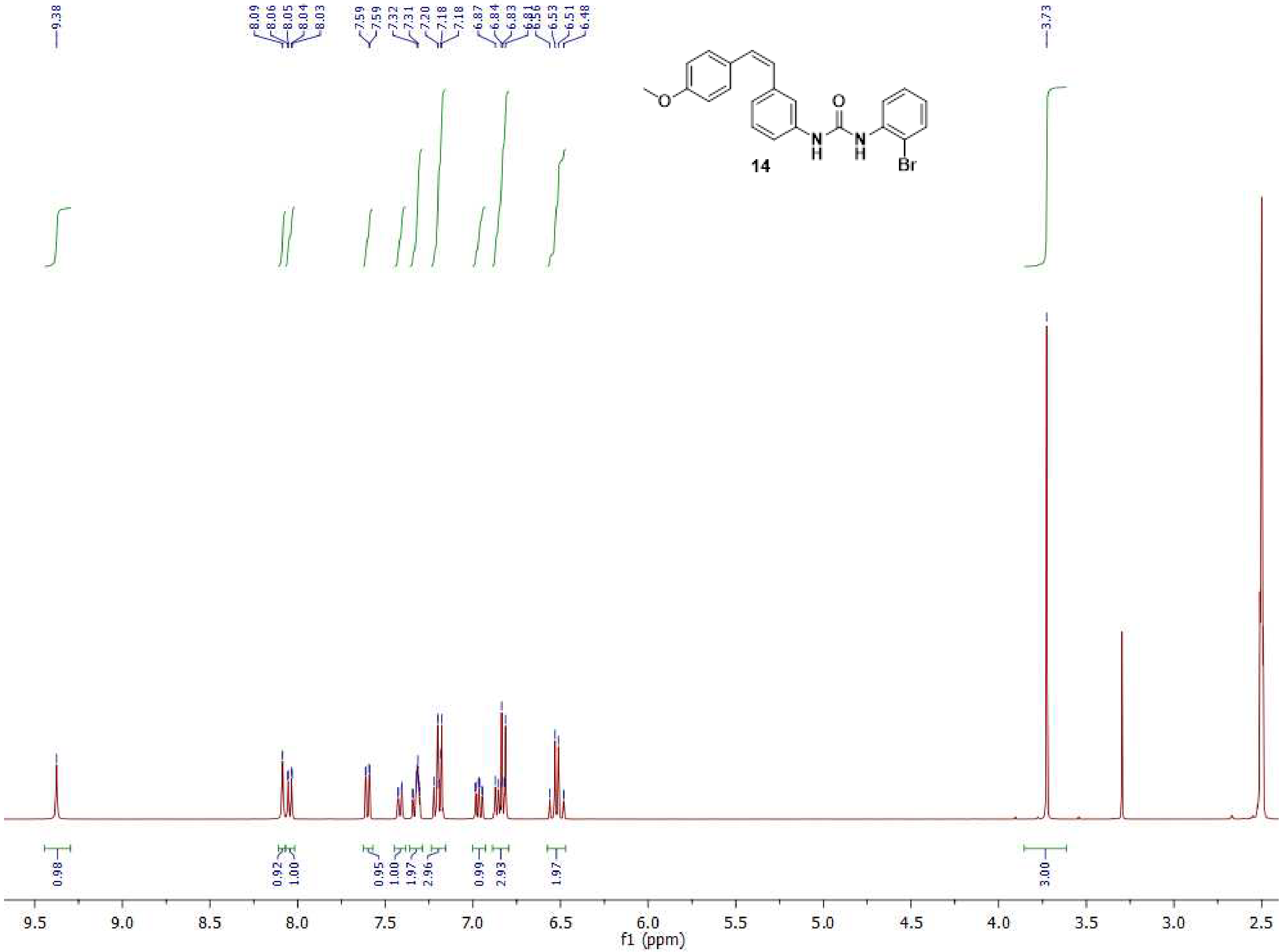

9.38
8.09
8.06
8.05
8.04
8.03
7.59
7.59
7.32
7.31
7.20
7.18
7.18
6.87
6.84
6.83
6.81
6.56
6.53
6.51
6.48
3.73
O
N
H
Br
14
0.98
0.92
1.00
0.95
1.00
1.97
2.96
0.99
2.93
1.97
3.00
9.5
9.0
8.5
8.0
7.5
7.0
6.5
6.0
5.5
5.0
4.5
4.0
3.5
3.0
2.5
f1 (ppm)

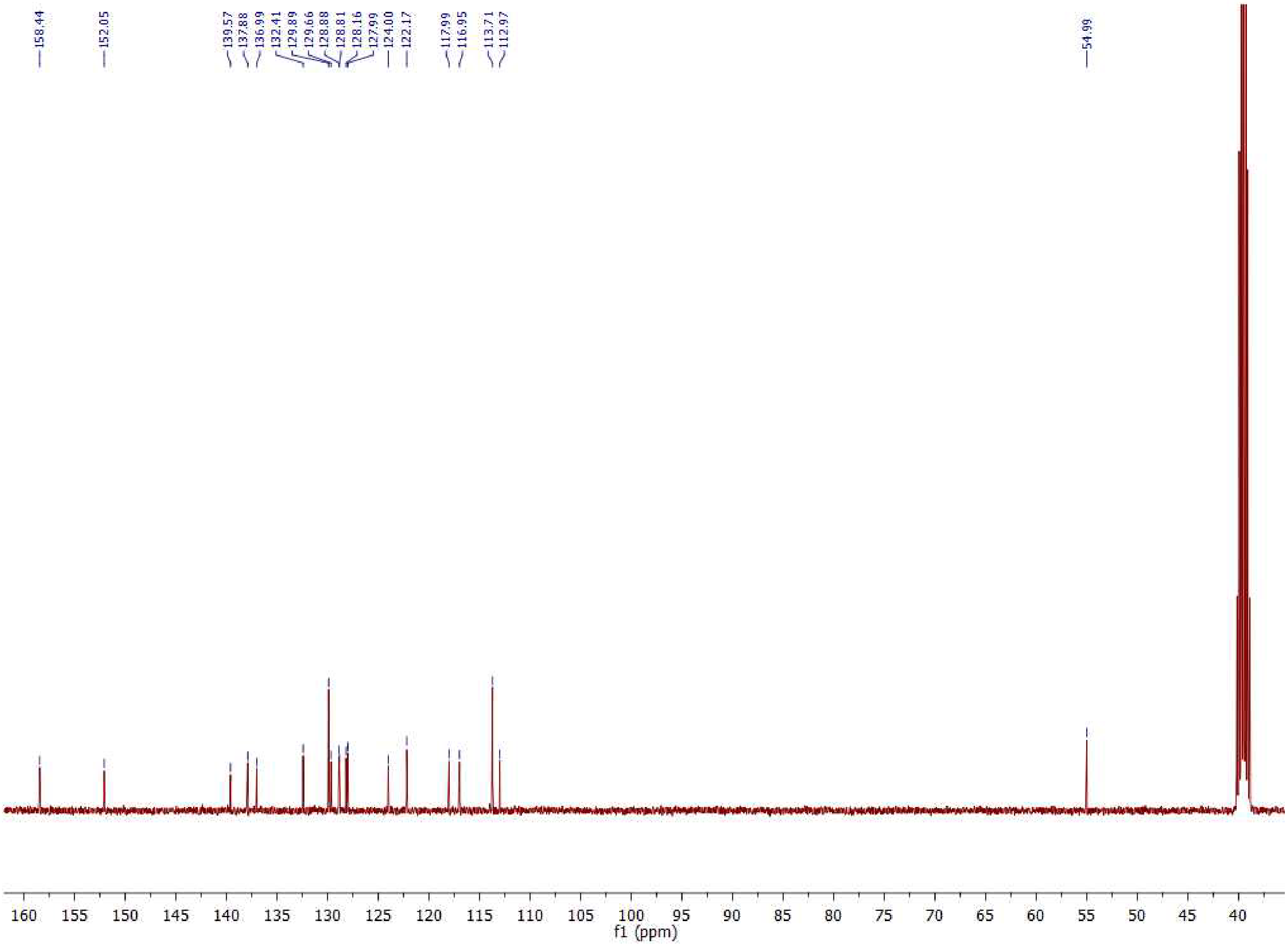
158.44
152.05
139.57
137.88
136.99
132.41
129.89
129.66
128.88
128.81
128.16
127.99
124.00
122.17
117.99
116.95
113.71
112.97
54.99
160 155 150 145 140 135 130 125 120 115 110 105 100 95 90 85 80 75 70 65 60 55 50 45 40
f1 (ppm)

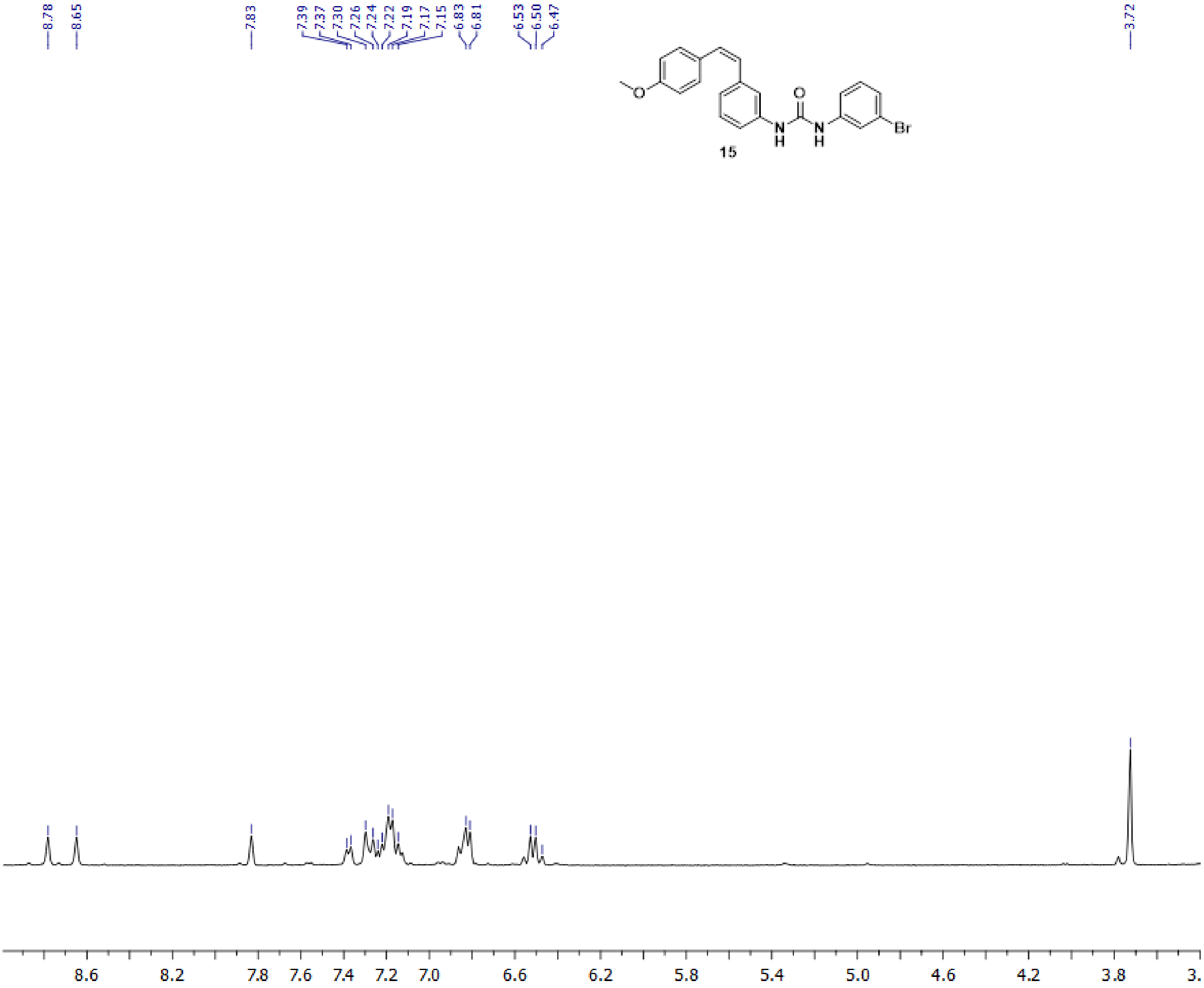
8.78
8.65
7.83
7.39
7.37
7.30
7.26
7.24
7.22
7.19
7.17
7.15
6.83
6.81
6.53
6.50
6.47
3.72
O
N
H
Br
15
8.6
8.2
7.8
7.6
7.4
7.2
7.0
6.6
6.2
5.8
5.4
5.0
4.6
4.2
3.8
3.

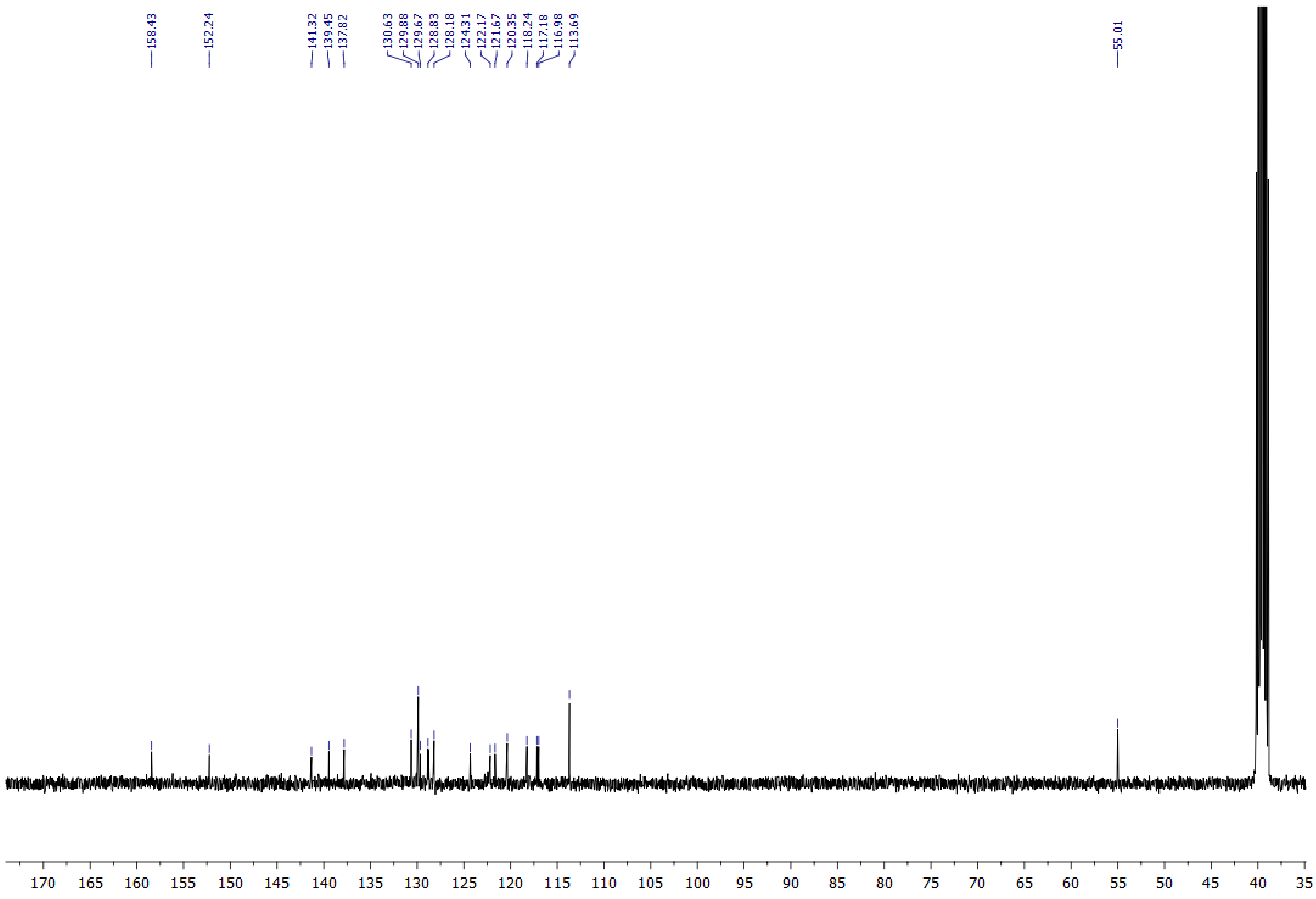
158.43
152.24
141.32
139.45
137.82
130.63
129.88
129.67
128.83
128.18
124.31
122.17
121.67
120.35
118.24
117.18
116.98
113.69
55.01
170 165 160 155 150 145 140 135 130 125 120 115 110 105 100 95 90 85 80 75 70 65 60 55 50 45 40 35

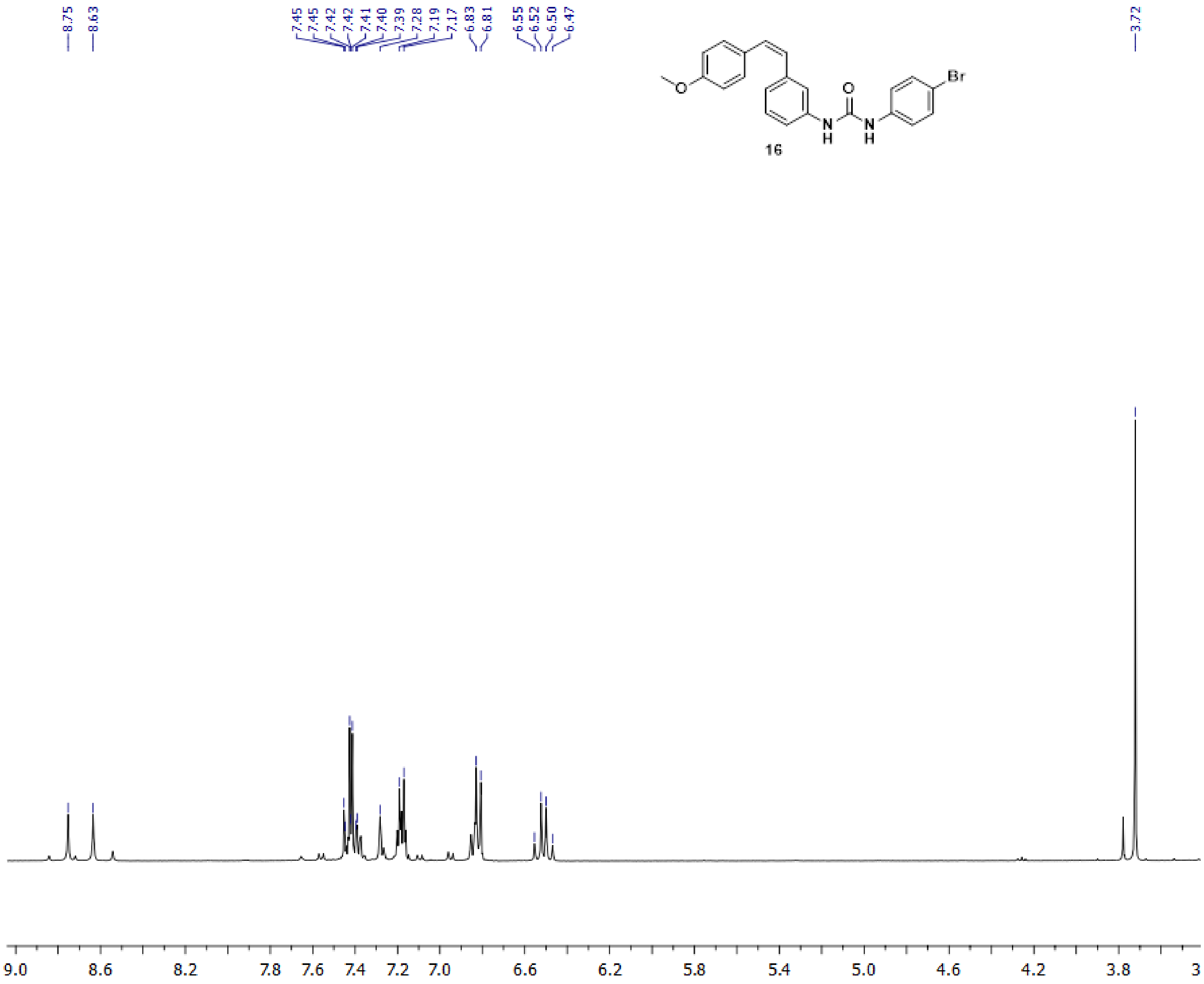
8.75
8.63
7.45
7.45
7.42
7.42
7.41
7.40
7.39
7.28
7.19
7.17
6.83
6.81
6.55
6.52
6.50
6.47
3.72
O
Br
N
H
N
H
16
9.0
8.6
8.2
7.8
7.6
7.4
7.2
7.0
6.6
6.2
5.8
5.4
5.0
4.6
4.2
3.8
3

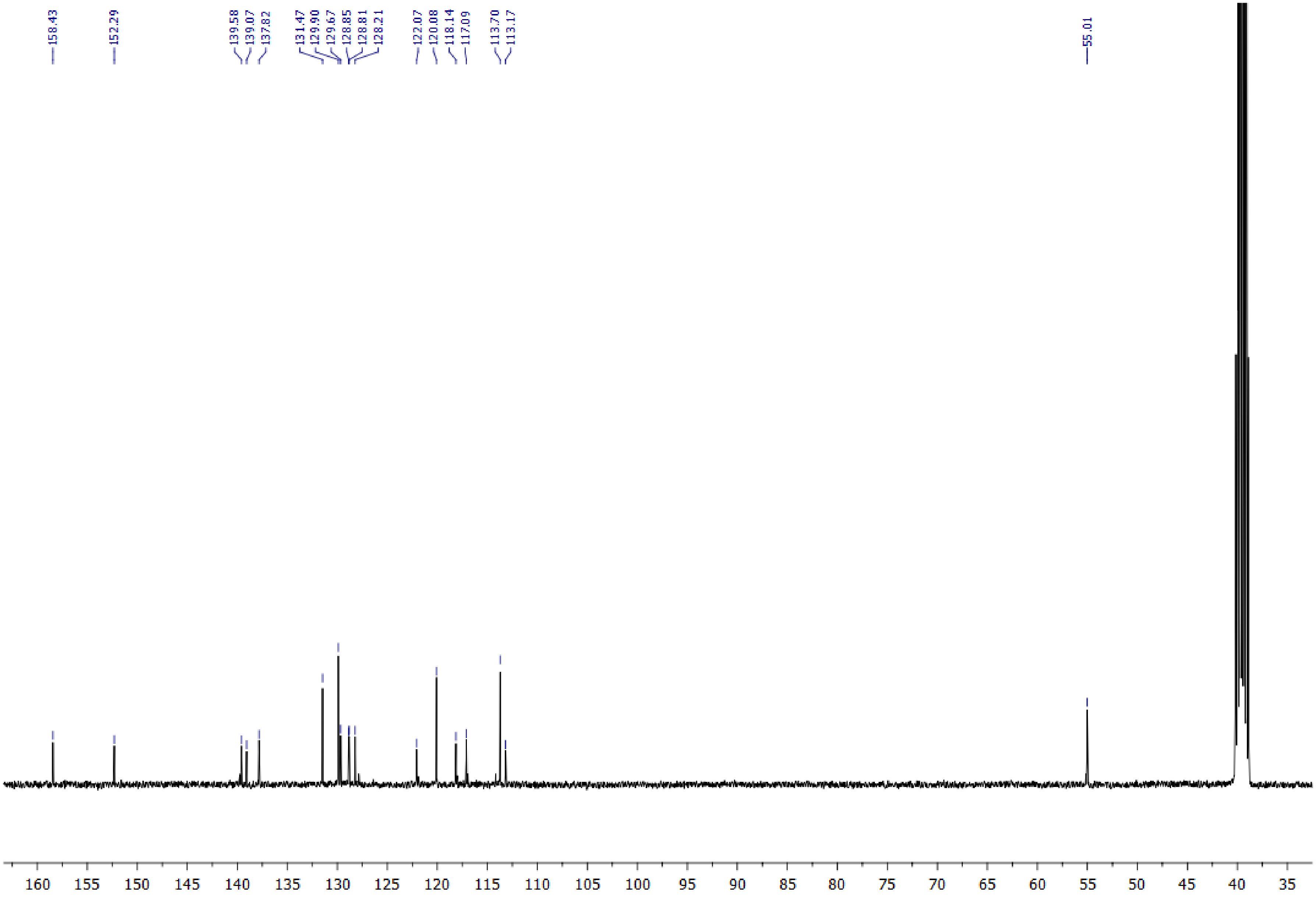
158.43
152.29
139.58
139.07
137.82
131.47
129.90
129.67
128.85
128.81
128.21
122.07
120.08
118.14
117.09
113.70
113.17
55.01
160 155 150 145 140 135 130 125 120 115 110 105 100 95 90 85 80 75 70 65 60 55 50 45 40 35

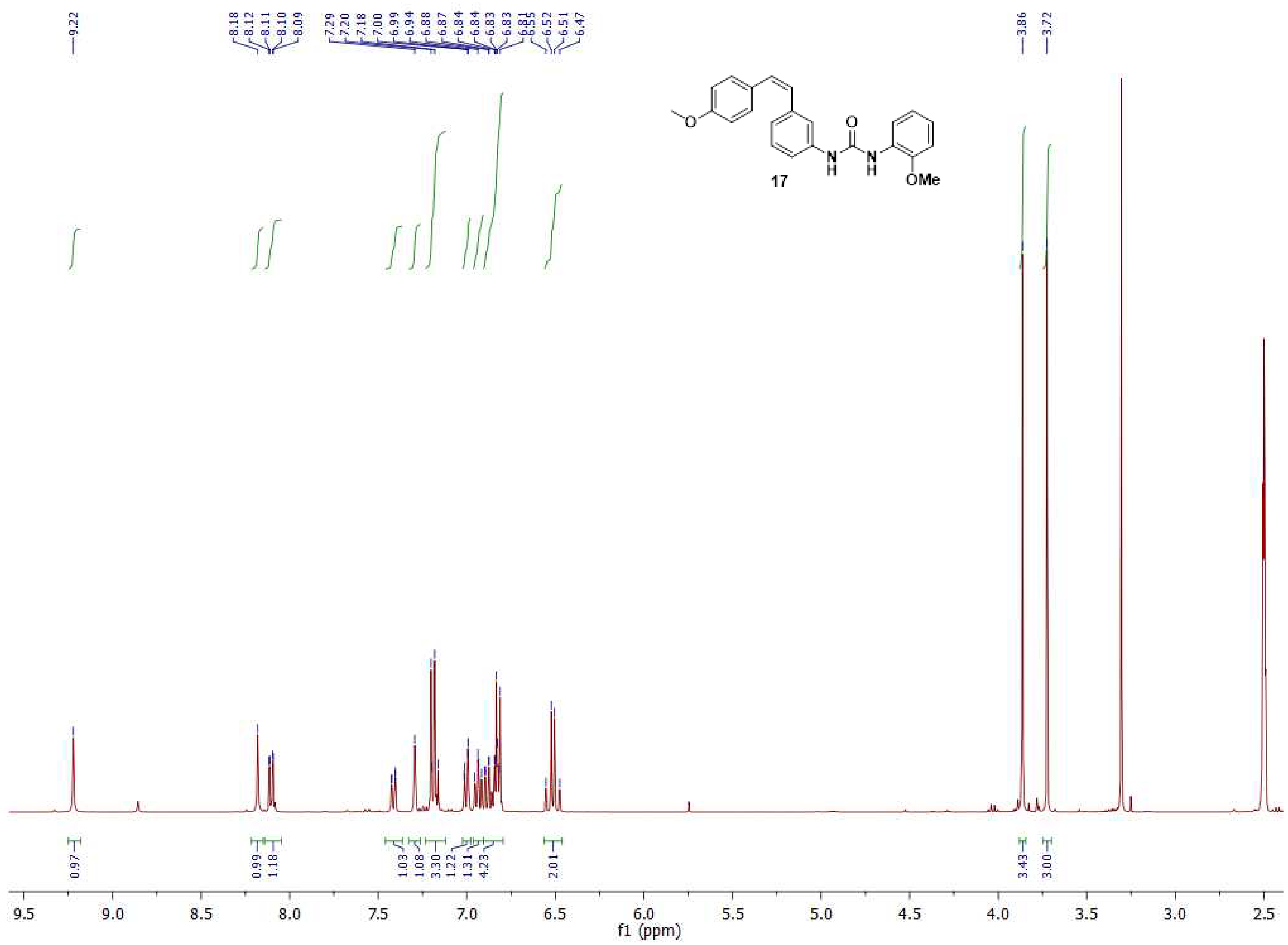
17
OMe
O
N
H
f1 (ppm)

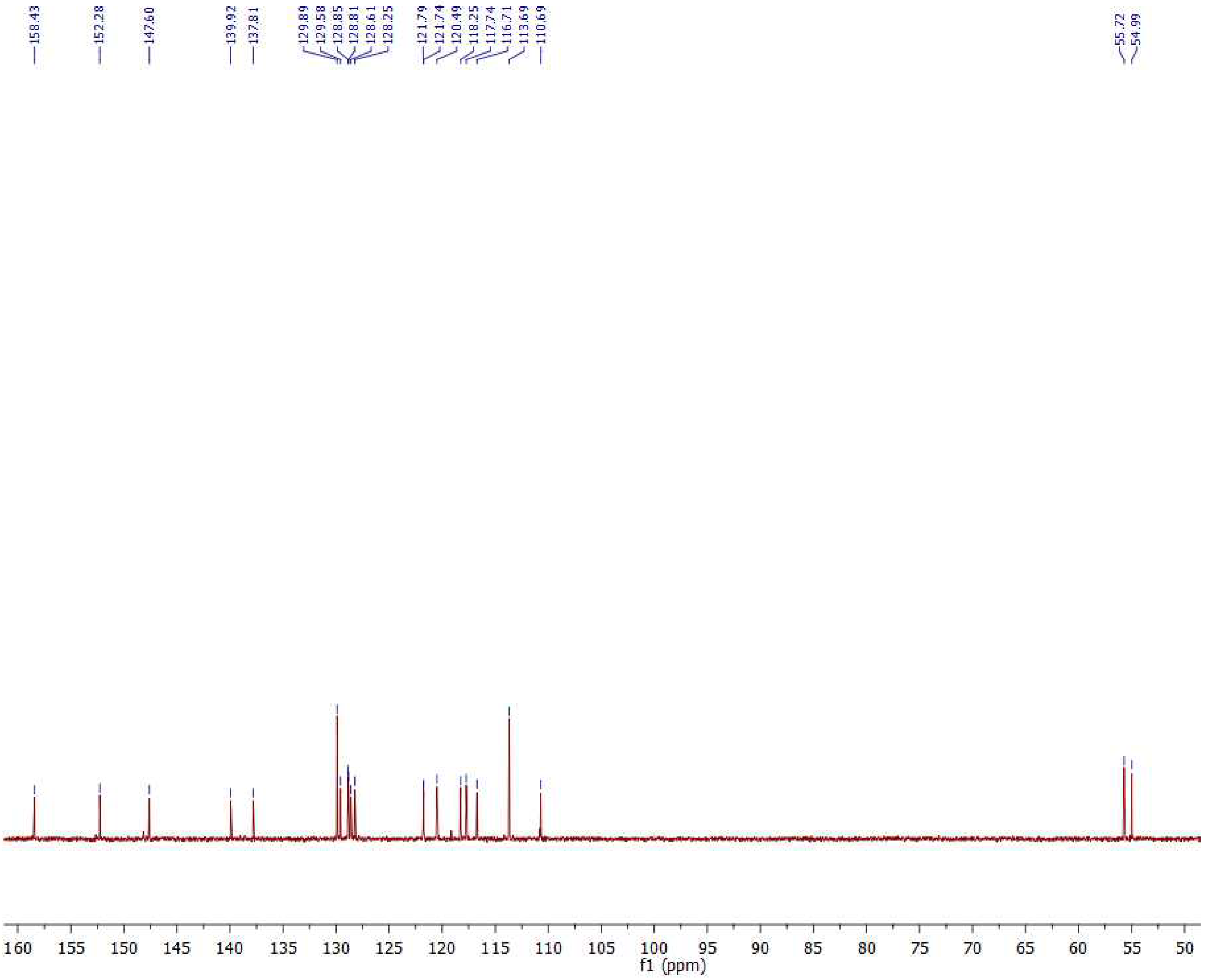
158.43
152.28
147.60
139.92
137.81
129.89
129.58
128.85
128.81
128.61
128.25
121.79
121.74
120.49
118.25
117.74
116.71
113.69
110.69
55.72
54.99
160 155 150 145 140 135 130 125 120 115 110 105 100 95 90 85 80 75 70 65 60 55 50
f1 (ppm)

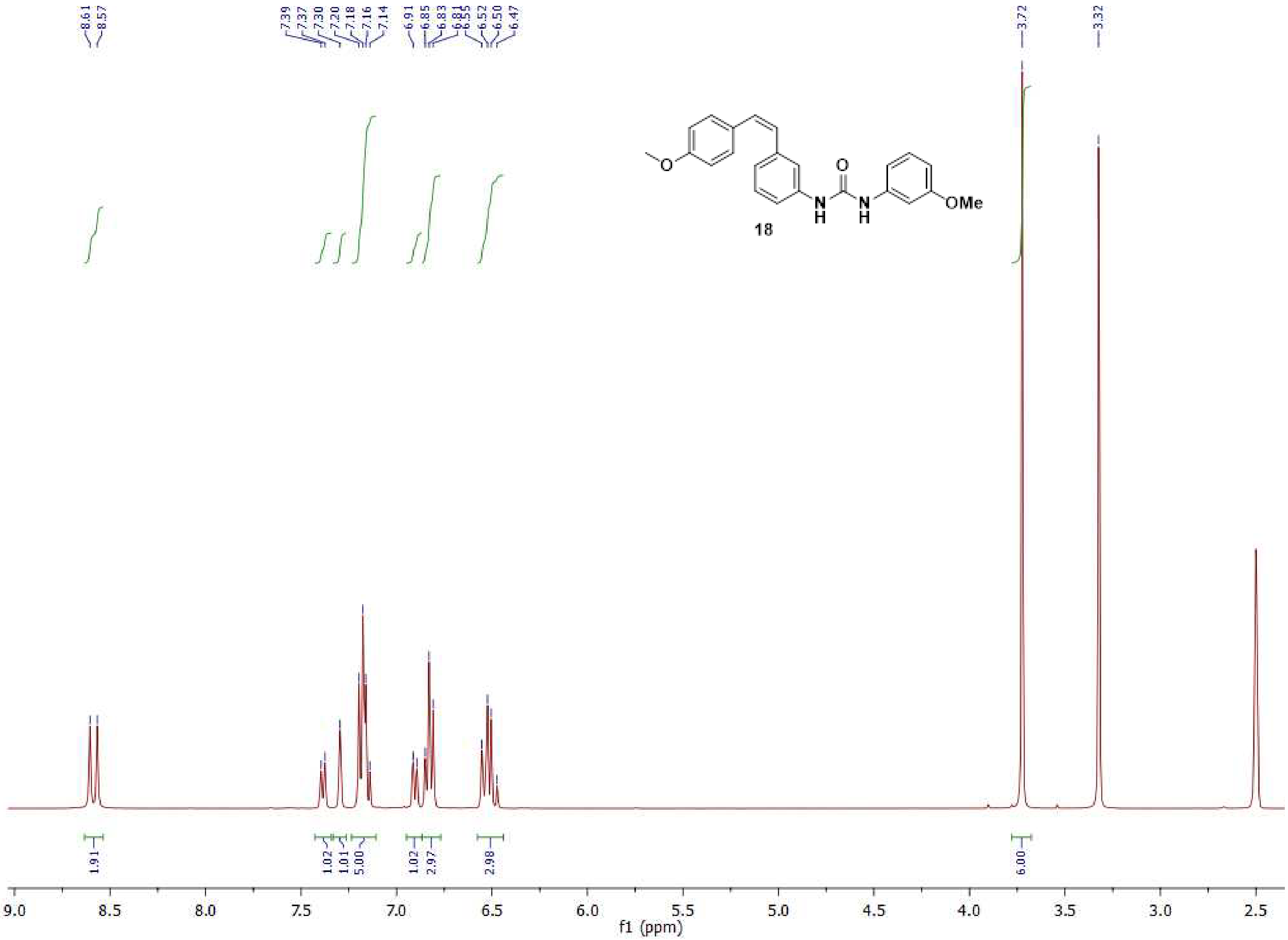
8.61
8.57
7.39
7.37
7.30
7.20
7.18
7.16
7.14
6.91
6.85
6.83
6.81
6.55
6.52
6.50
6.47
3.72
3.32
OMe
O
N
H
N
H
18
1.91
1.02
1.01
5.00
1.02
2.97
2.98
6.00
9.0
8.5
8.0
7.5
7.0
6.5
6.0
5.5
5.0
4.5
4.0
3.5
3.0
2.5
f1 (ppm)

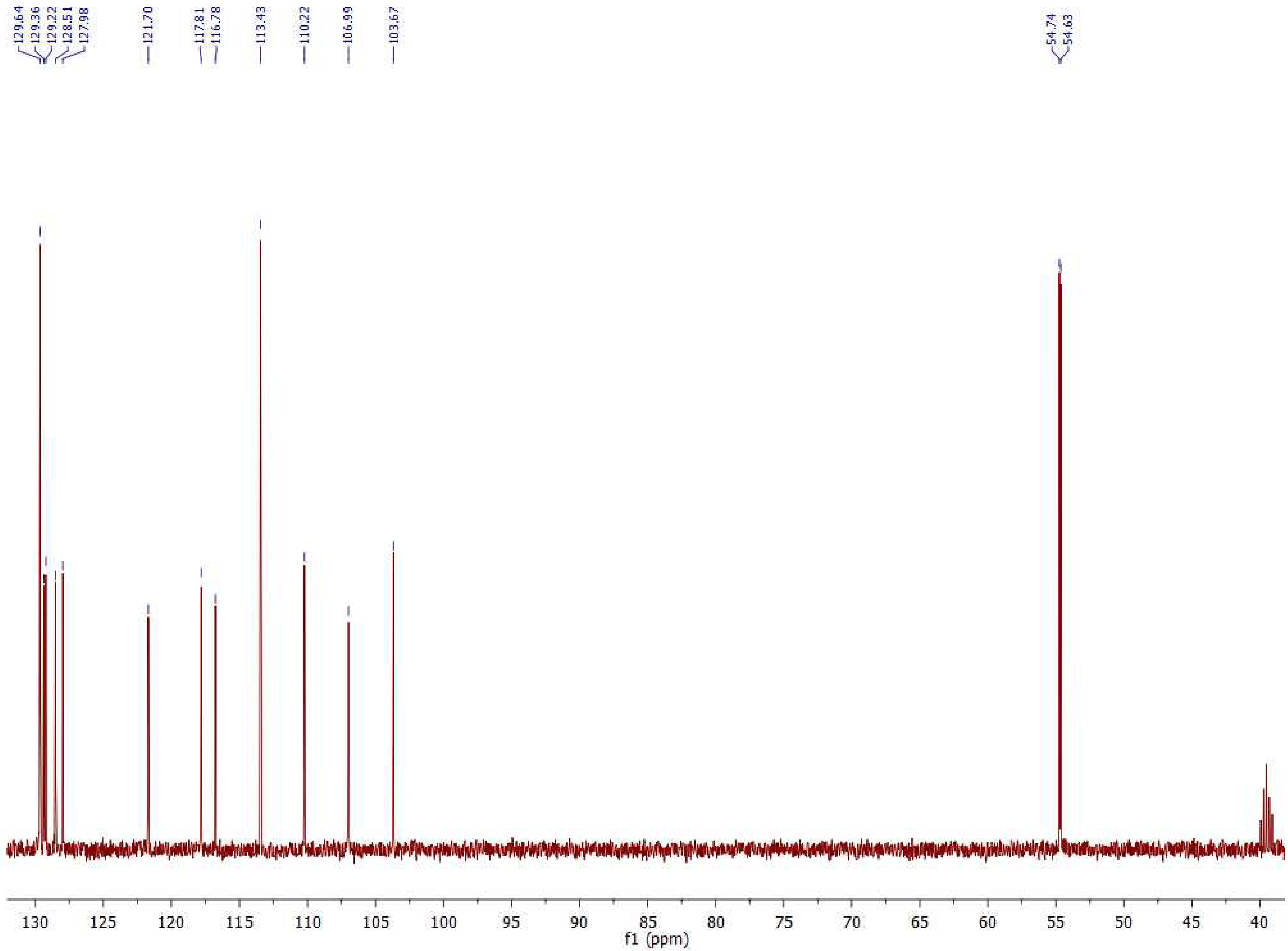
129.64
129.36
129.22
128.51
127.98
121.70
117.81
116.78
113.43
110.22
106.99
103.67
54.74
54.63
130
125
120
115
110
105
100
95
90
85
80
75
70
65
60
55
50
45
40
f1 (ppm)

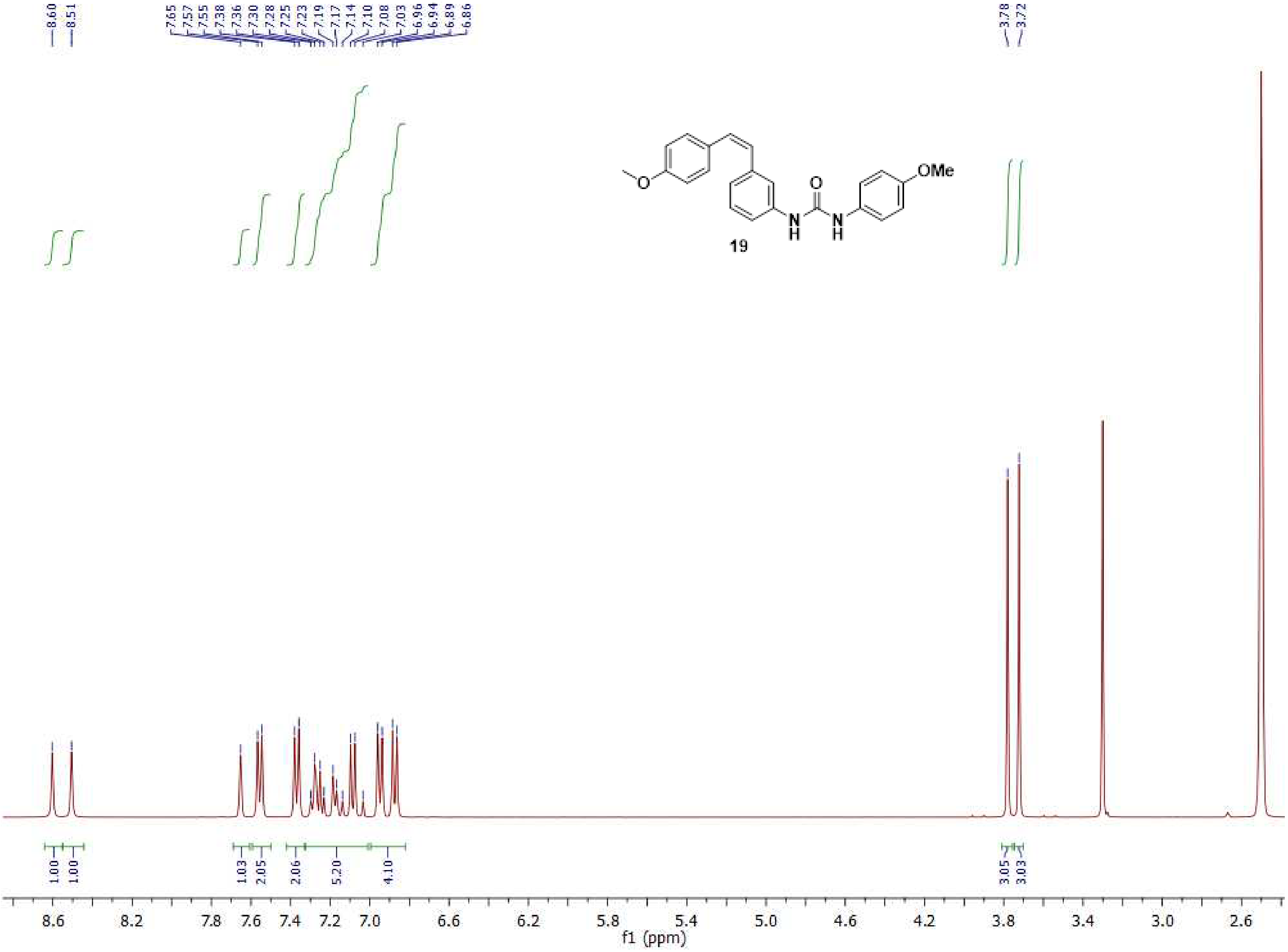

8.60
8.51
7.65
7.57
7.55
7.38
7.36
7.30
7.28
7.25
7.23
7.19
7.17
7.14
7.10
7.08
7.03
6.96
6.94
6.89
6.86
3.78
3.72
OMe
O
O
N
H
N
H
19
1.00
1.00
1.03
2.05
2.06
5.20
4.10
3.05
3.03
f1 (ppm)

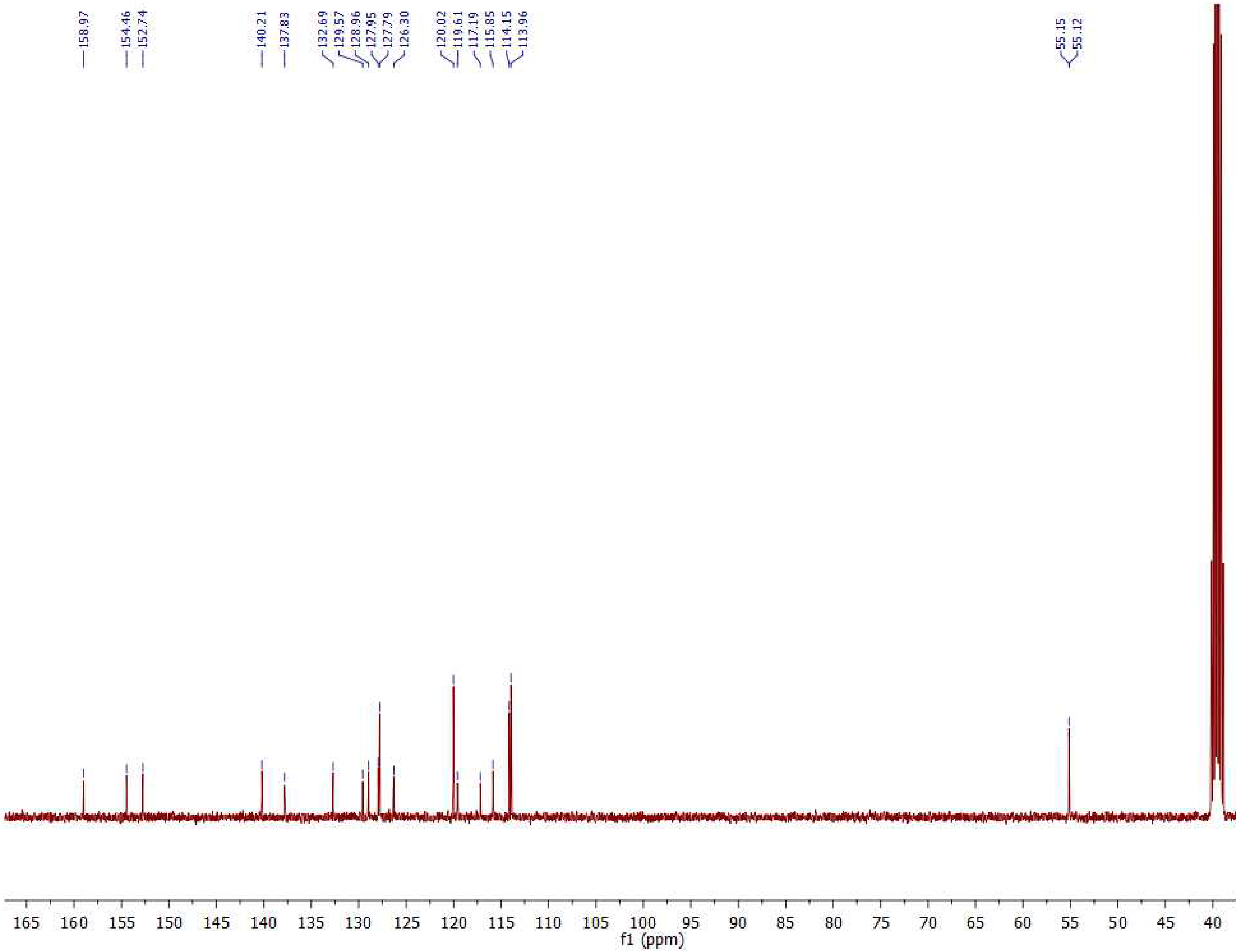
158.97
154.46
152.74
140.21
137.83
132.69
129.57
128.96
127.95
127.79
126.30
120.02
119.61
117.19
115.85
114.15
113.96
55.15
55.12
165 160 155 150 145 140 135 130 125 120 115 110 105 100 95 90 85 80 75 70 65 60 55 50 45 40
f1 (ppm)

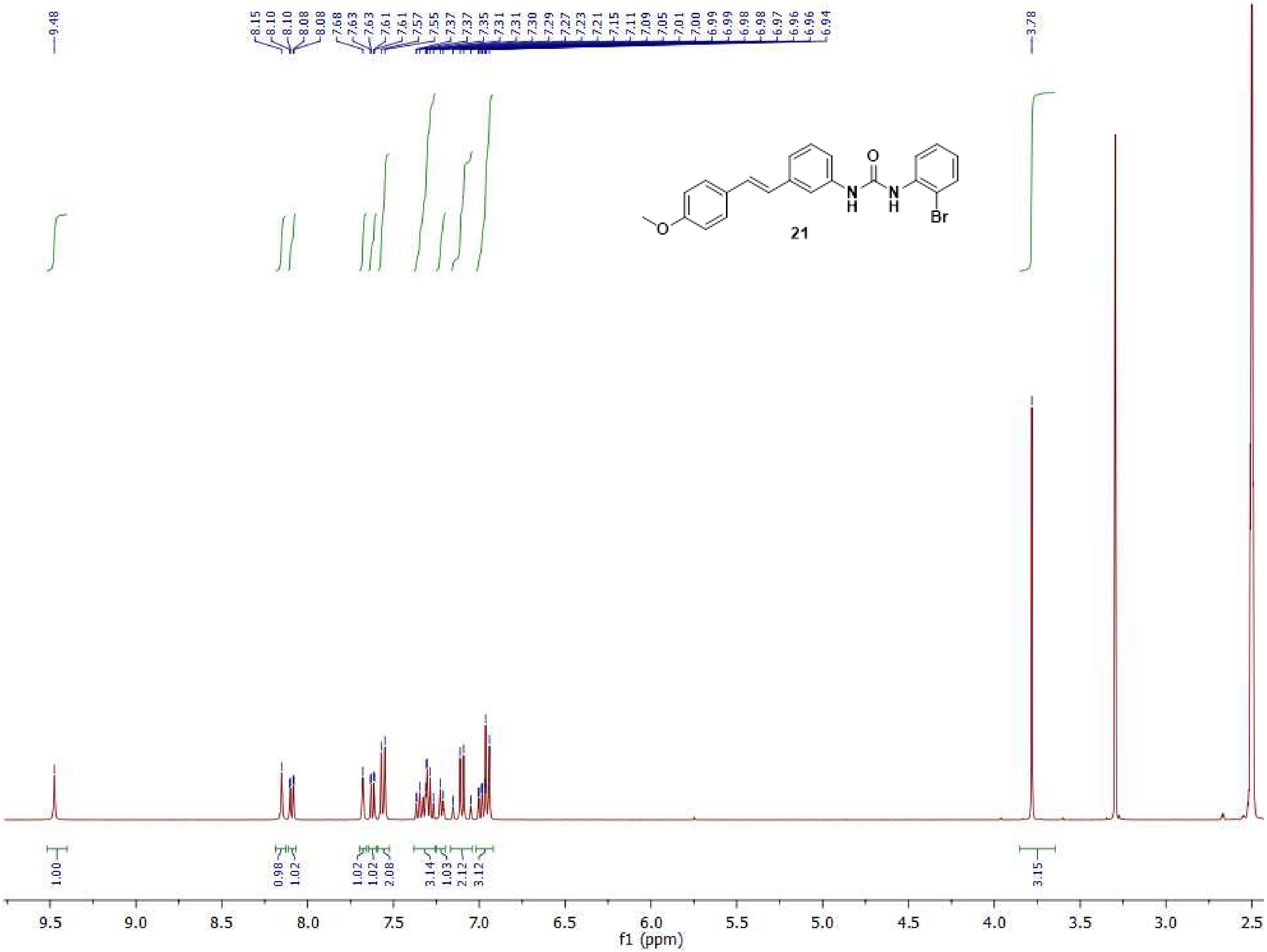
9.48
8.15
8.10
8.10
8.08
8.08
7.68
7.63
7.63
7.61
7.61
7.57
7.55
7.37
7.37
7.35
7.31
7.31
7.30
7.29
7.27
7.23
7.21
7.15
7.11
7.09
7.05
7.01
7.00
6.99
6.99
6.98
6.98
6.97
6.96
6.96
6.94
3.78
O
N
H
N
H
O
Br
21
1.00
0.98
1.02
1.02
1.02
2.08
3.14
1.03
2.12
3.12
3.15
9.5
9.0
8.5
8.0
7.5
7.0
6.5
6.0
5.5
5.0
4.5
4.0
3.5
3.0
2.5
f1 (ppm)

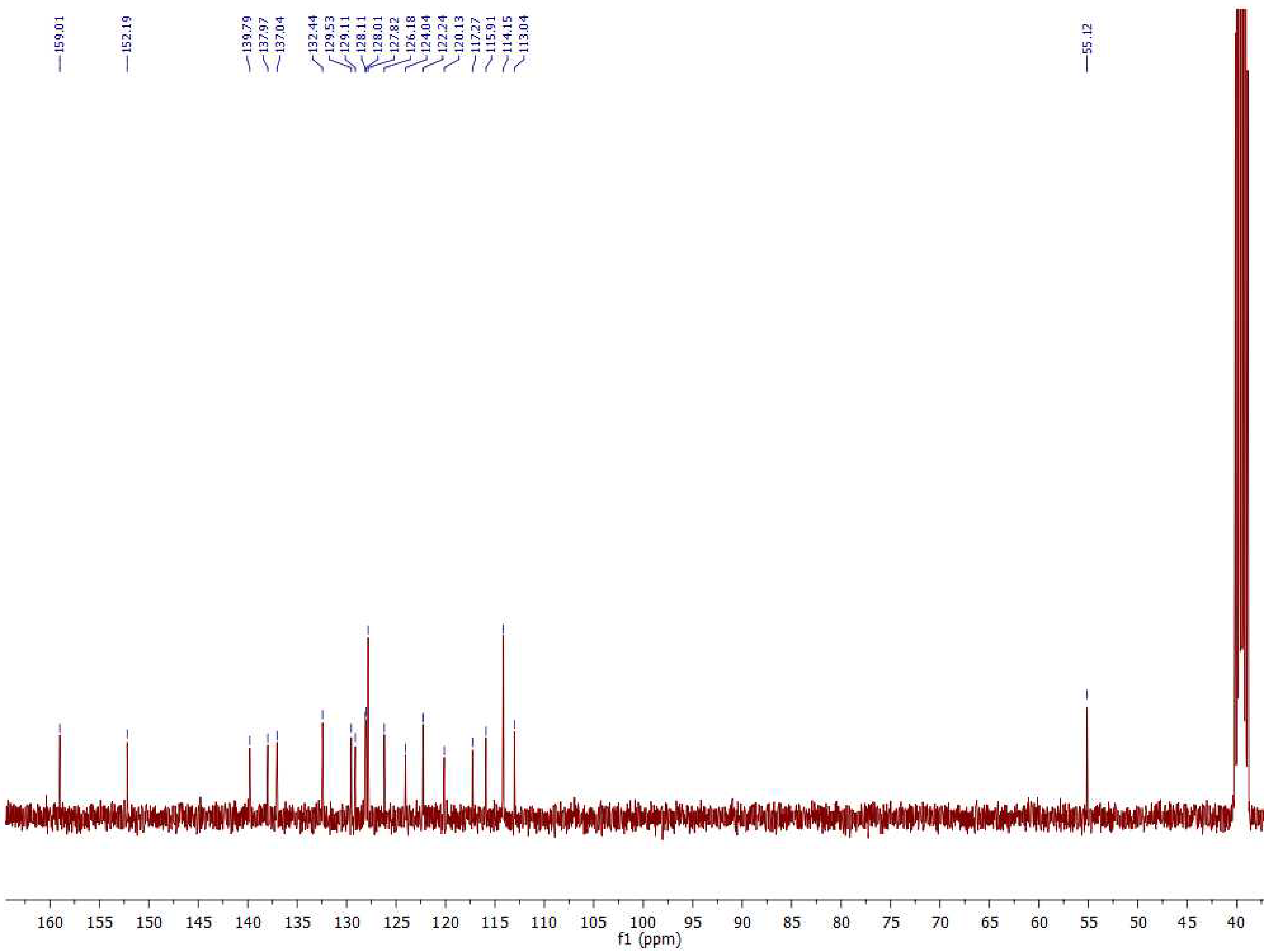
159.01
152.19
139.79
137.97
137.04
132.44
129.53
129.11
128.11
128.01
127.82
126.18
124.04
122.24
120.13
117.27
115.91
114.15
113.04
55.12
160 155 150 145 140 135 130 125 120 115 110 105 100 95 90 85 80 75 70 65 60 55 50 45 40
f1 (ppm)

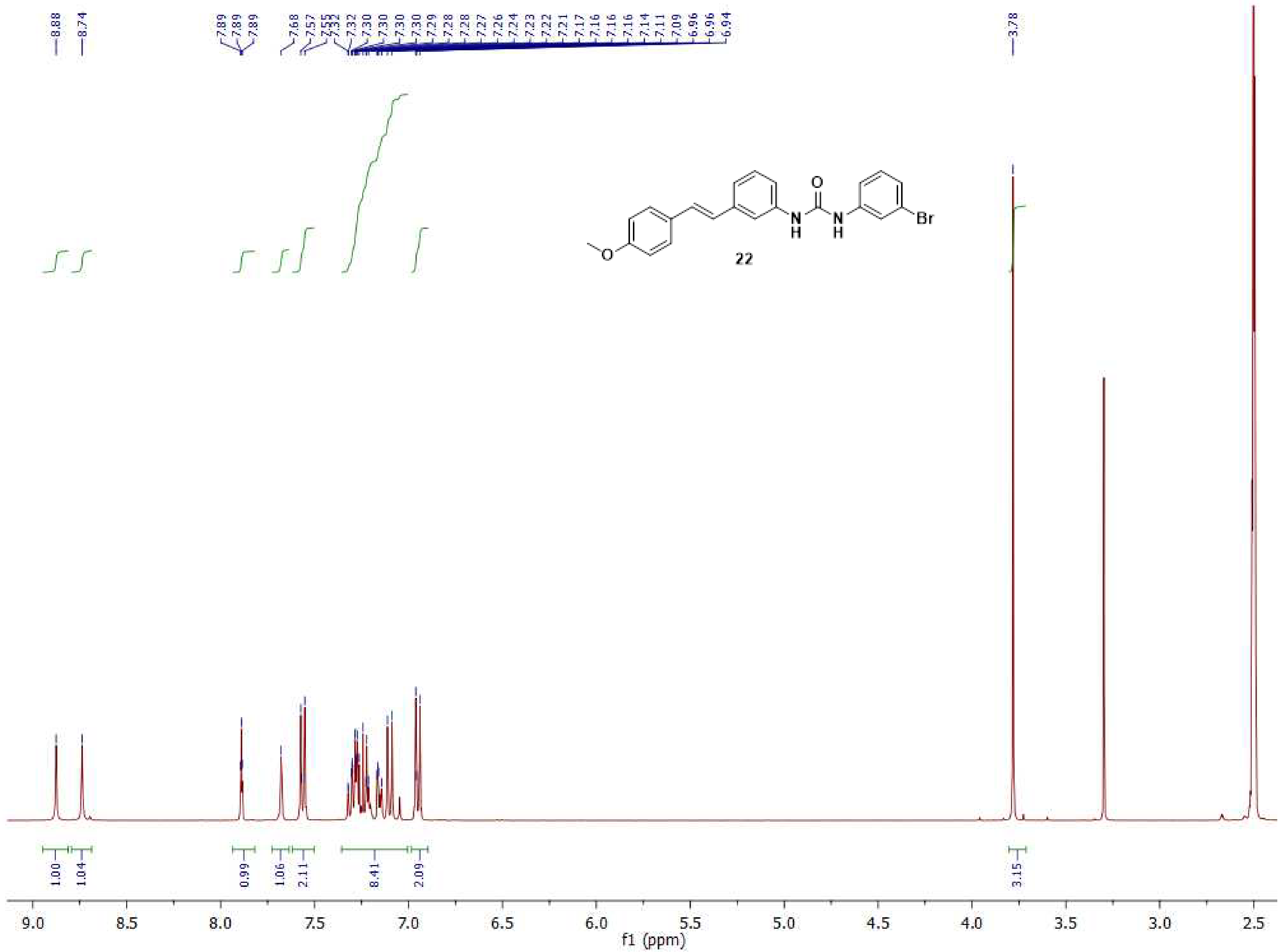
8.88
8.74
7.89
7.89
7.89
7.68
7.57
7.55
7.32
7.32
7.30
7.30
7.30
7.30
7.29
7.28
7.28
7.27
7.26
7.24
7.23
7.22
7.21
7.17
7.16
7.16
7.16
7.14
7.11
7.09
6.96
6.96
6.94
3.78
O
N
H
N
H
Br
O
22
1.00
1.04
0.99
1.06
2.11
8.41
2.09
3.15
9.0
8.5
8.0
7.5
7.0
6.5
6.0
5.5
5.0
4.5
4.0
3.5
3.0
2.5
f1 (ppm)

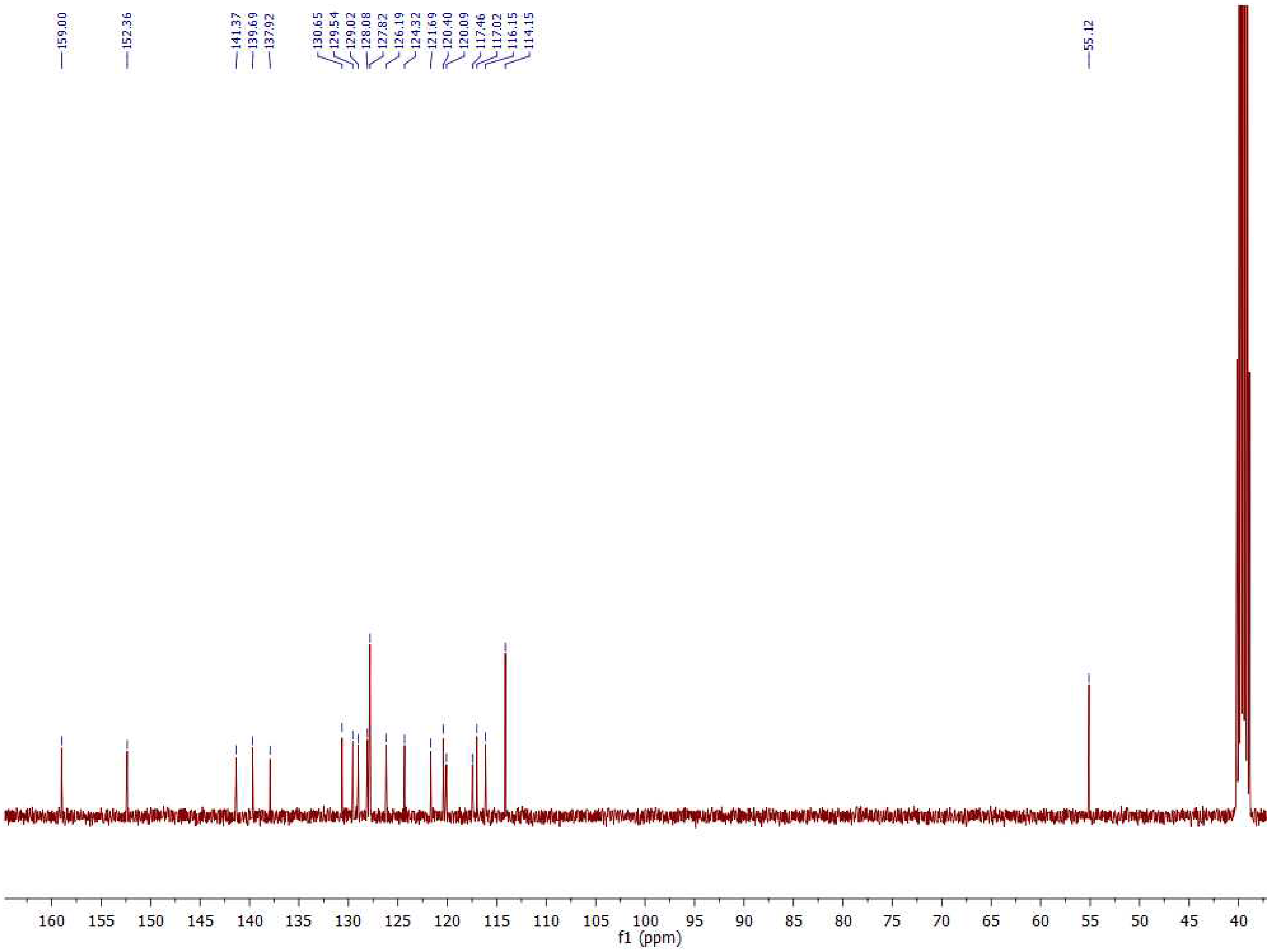
159.00
152.36
141.37
139.69
137.92
130.65
129.54
129.02
128.08
127.82
126.19
124.32
121.69
120.40
120.09
117.46
117.02
116.15
114.15
55.12
160 155 150 145 140 135 130 125 120 115 110 105 100 95 90 85 80 75 70 65 60 55 50 45 40
f1 (ppm)

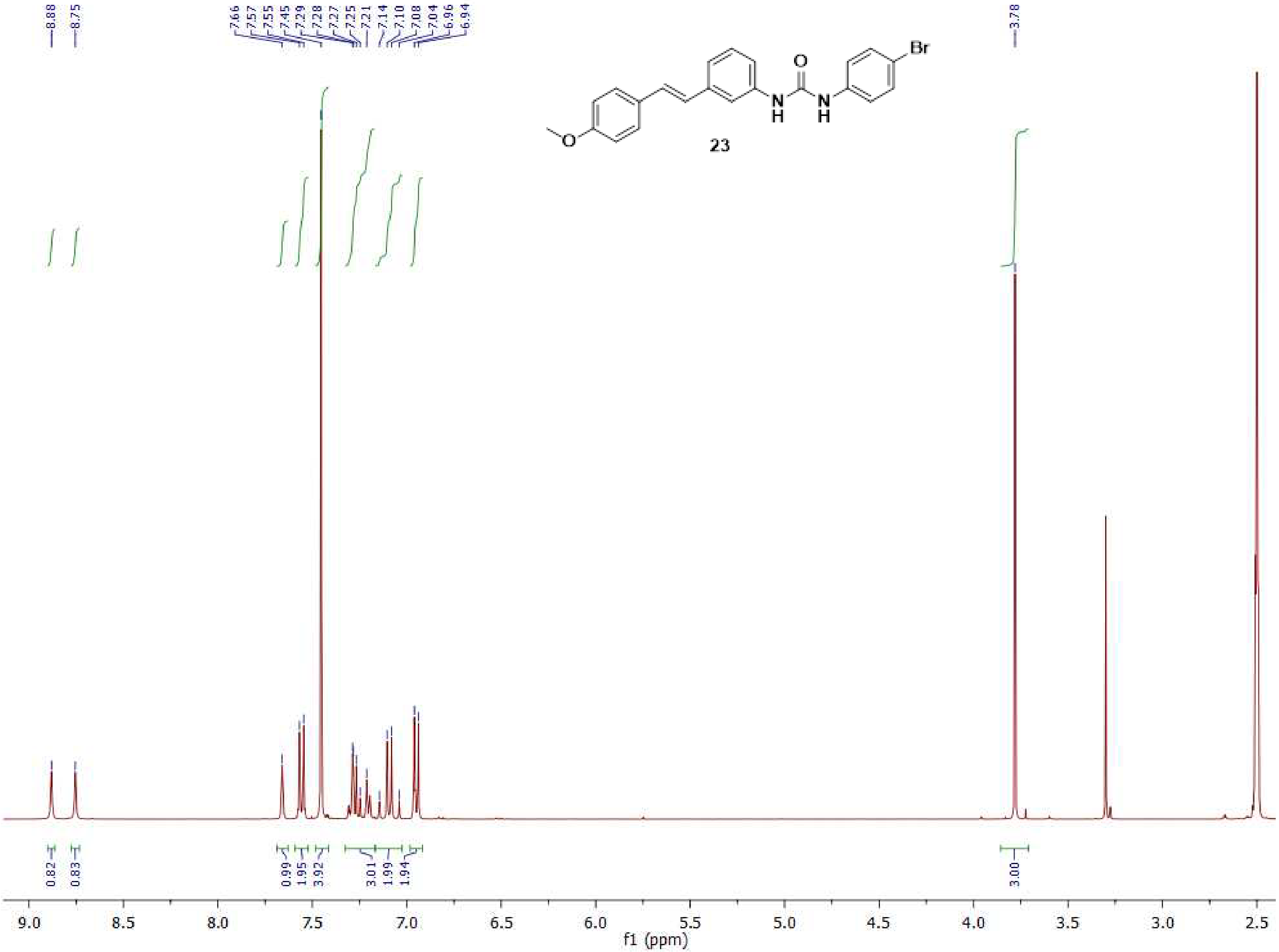
8.88
8.75
7.66
7.57
7.55
7.45
7.29
7.28
7.27
7.25
7.21
7.14
7.10
7.08
7.04
6.96
6.94
3.78
O
Br
N
H
N
H
O
23
0.82
0.83
0.99
1.95
3.92
3.01
1.99
1.94
3.00
9.0
8.5
8.0
7.5
7.0
6.5
6.0
5.5
5.0
4.5
4.0
3.5
3.0
2.5
f1 (ppm)

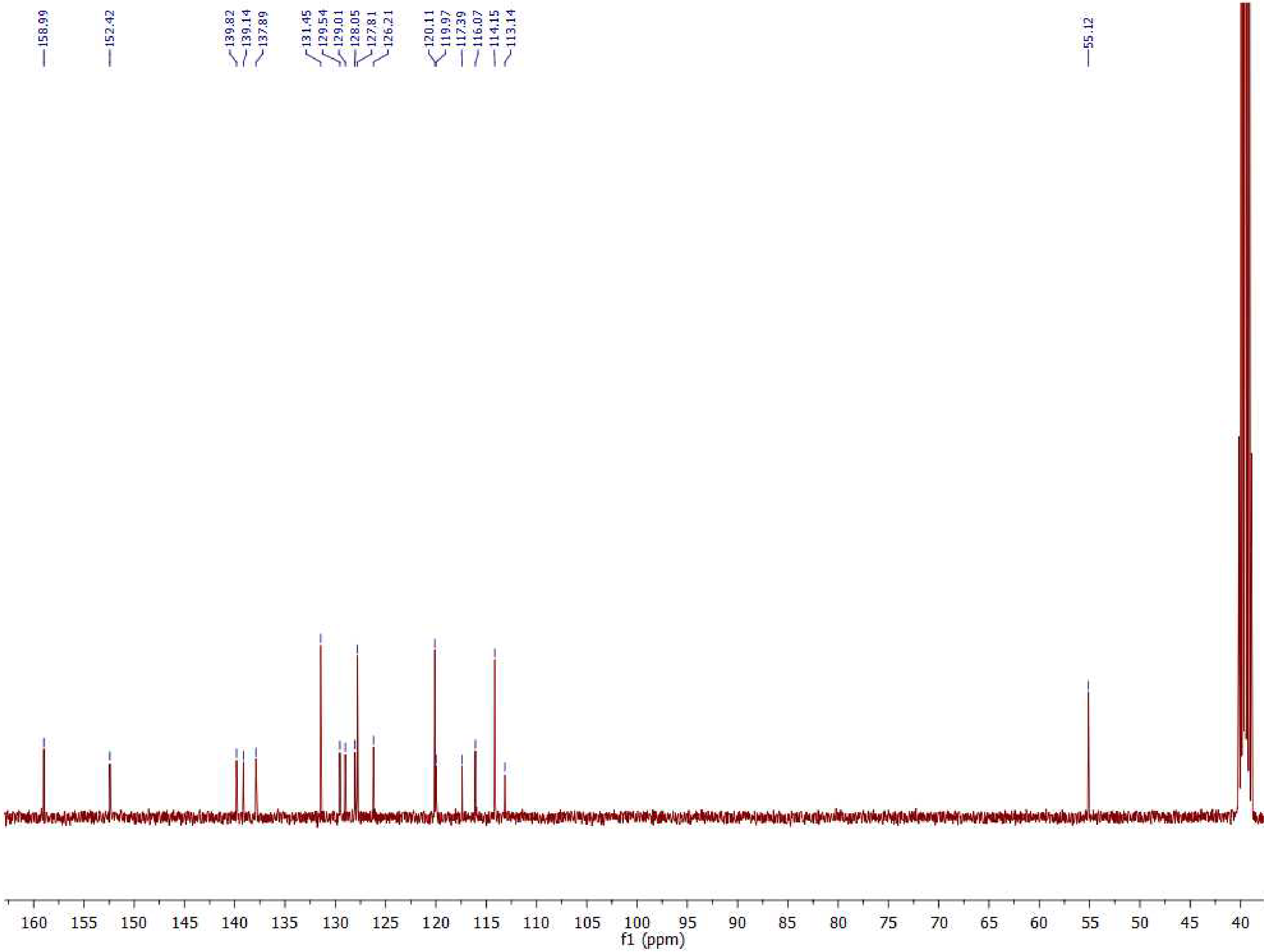
158.99
152.42
139.82
139.14
137.89
131.45
129.54
129.01
128.05
127.81
126.21
120.11
119.97
117.39
116.07
114.15
113.14
55.12
160 155 150 145 140 135 130 125 120 115 110 105 100 95 90 85 80 75 70 65 60 55 50 45 40
f1 (ppm)

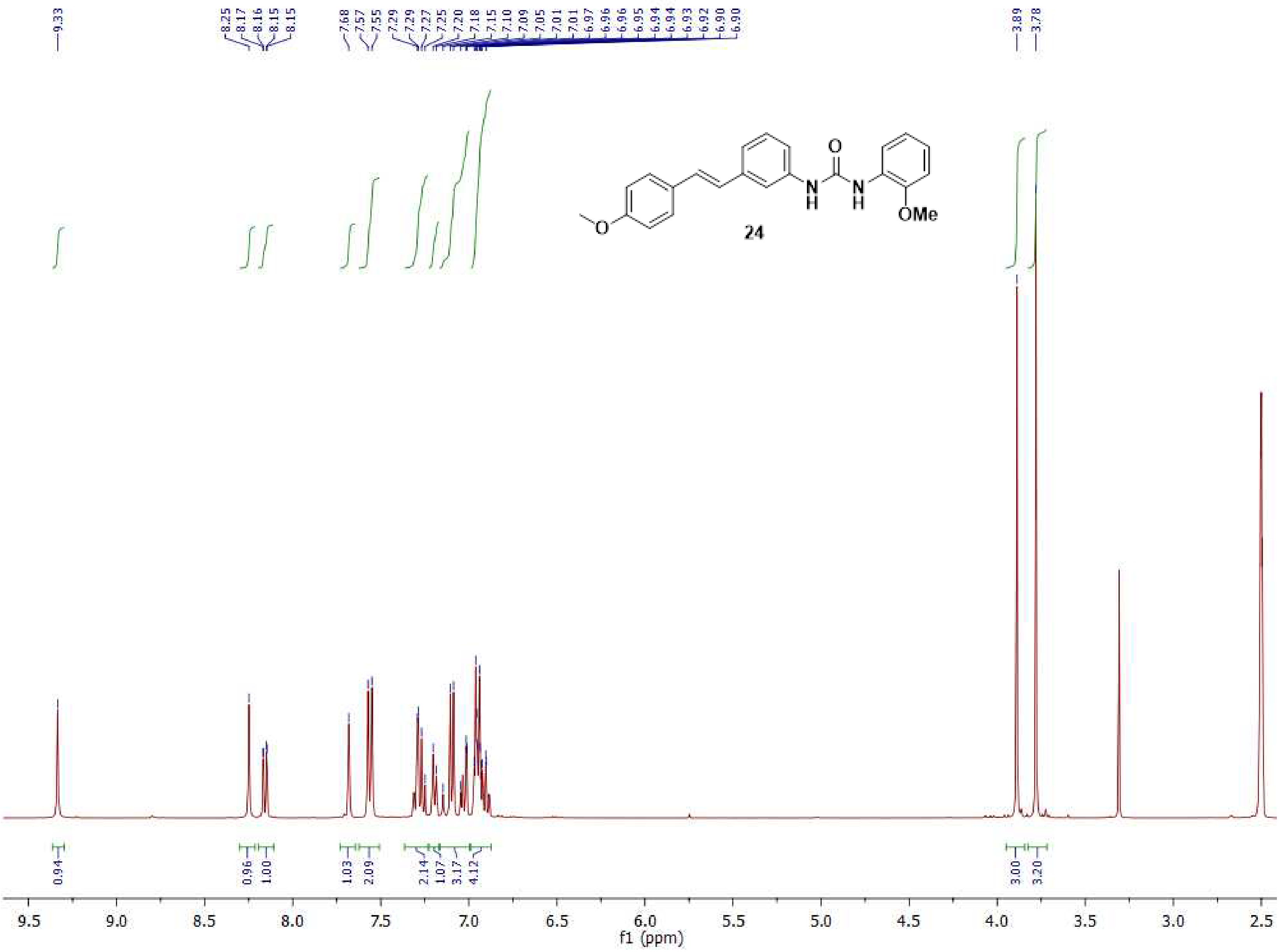
24
OMe
f1 (ppm)

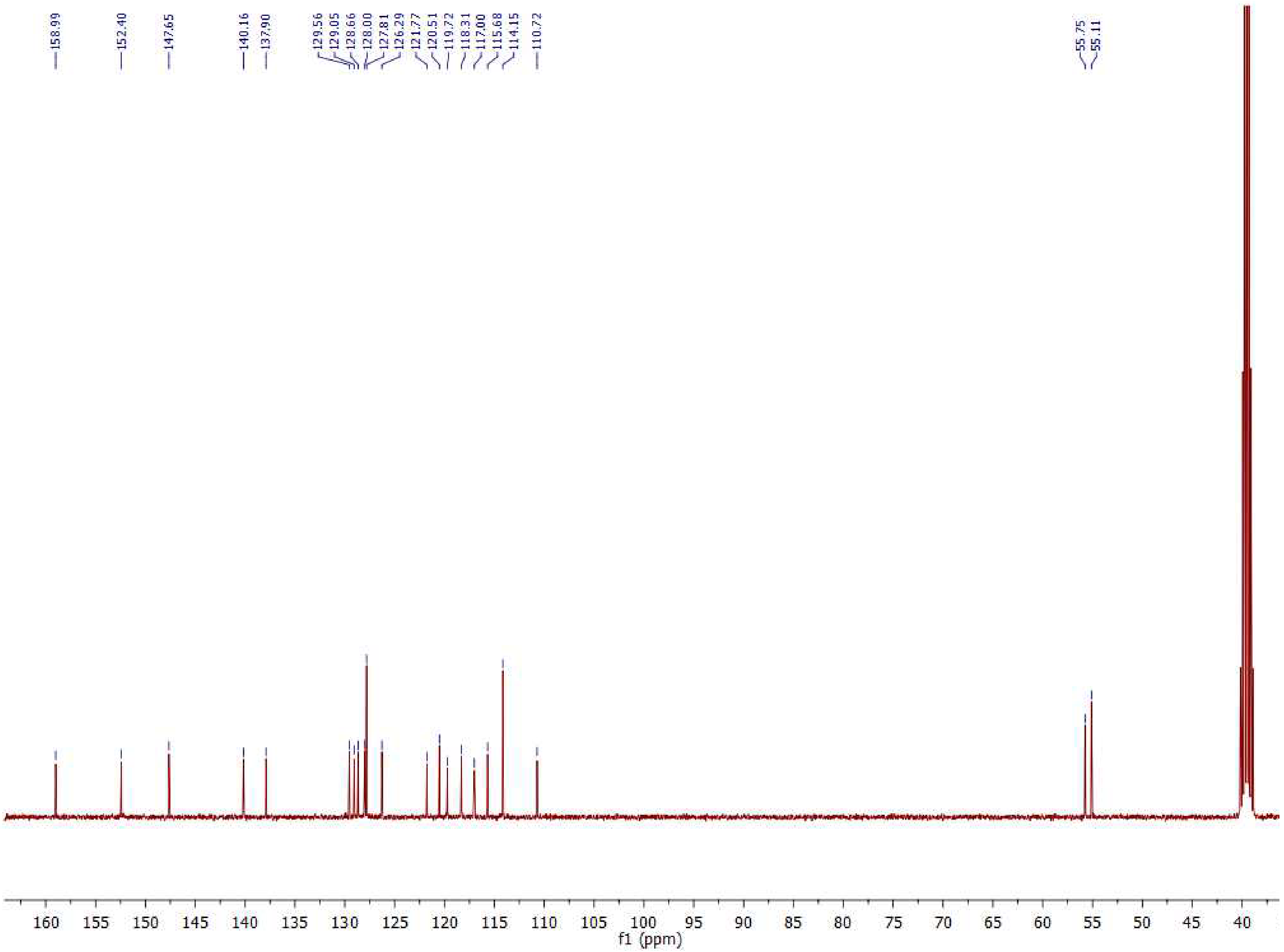
158.99
152.40
147.65
140.16
137.90
129.56
129.05
128.66
128.00
127.81
126.29
121.77
120.51
119.72
118.31
117.00
115.68
114.15
110.72
55.75
55.11
160 155 150 145 140 135 130 125 120 115 110 105 100 95 90 85 80 75 70 65 60 55 50 45 40
f1 (ppm)

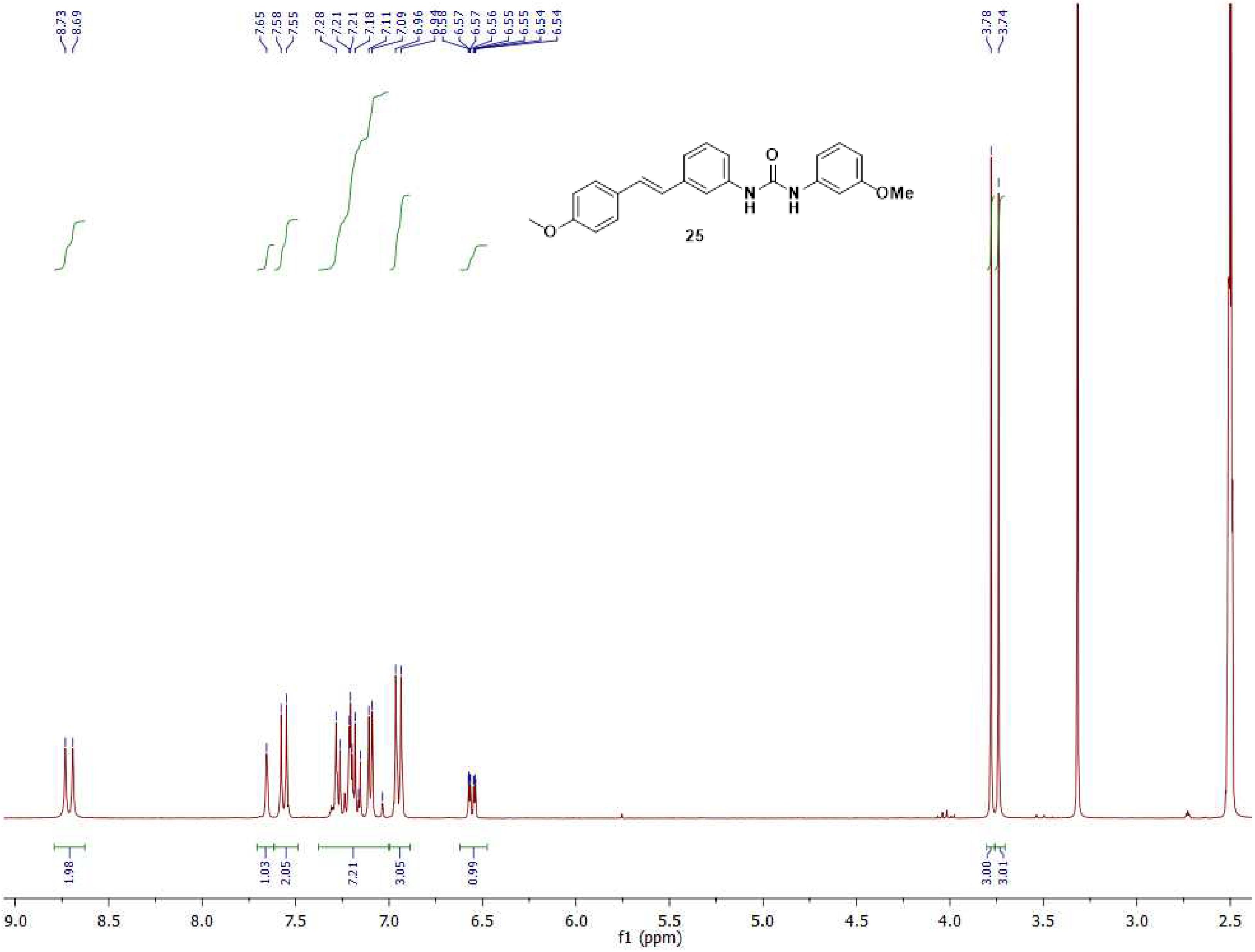
8.73
8.69
7.65
7.58
7.55
7.28
7.21
7.21
7.18
7.11
7.09
6.96
6.94
6.58
6.57
6.57
6.56
6.55
6.55
6.54
6.54
3.78
3.74
N
H
O
N
H
OMe
O
25
1.98
1.03
2.05
7.21
3.05
0.99
3.00
3.01
9.0
8.5
8.0
7.5
7.0
6.5
6.0
5.5
5.0
4.5
4.0
3.5
3.0
2.5
f1 (ppm)

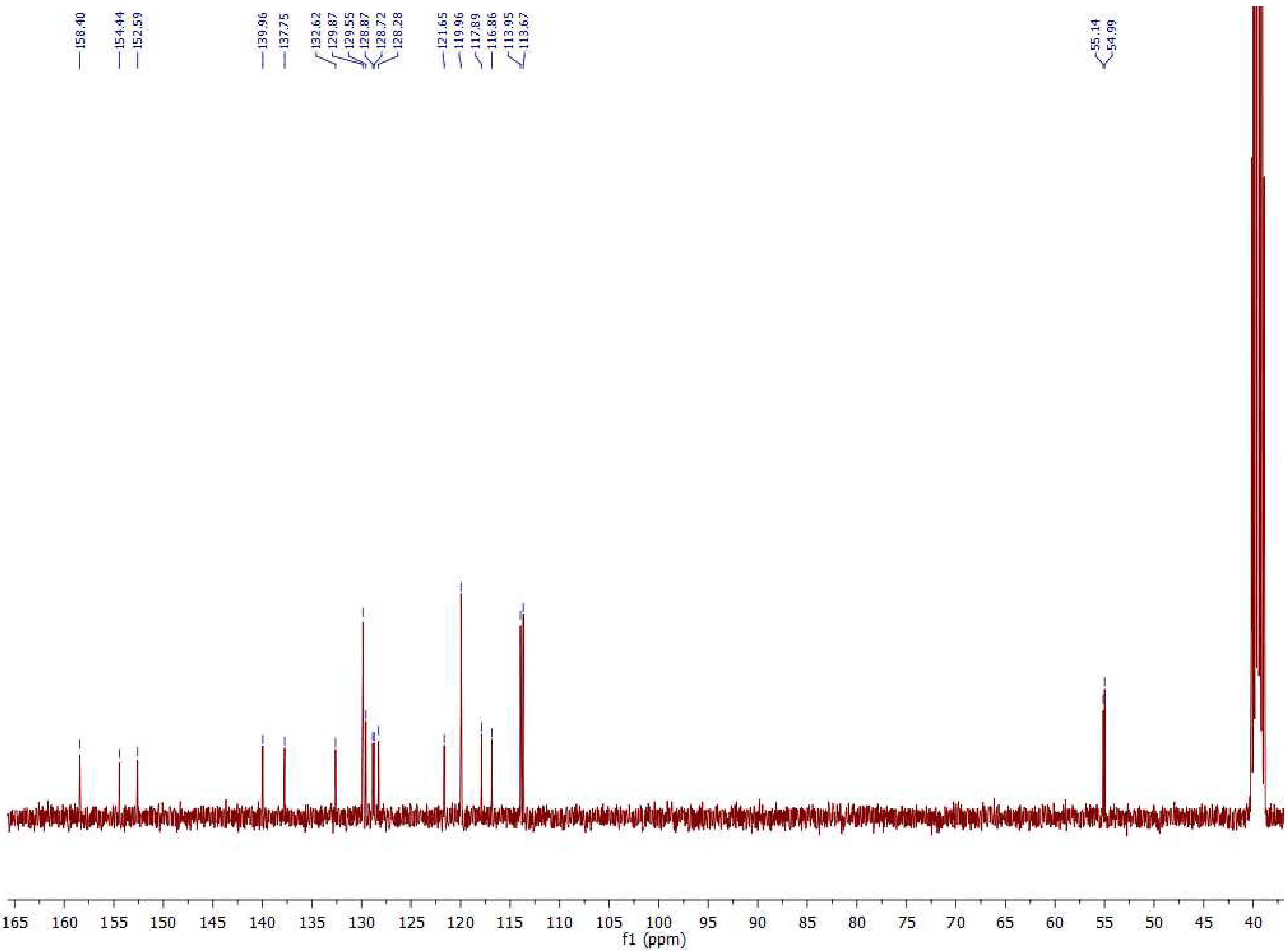
158.40
154.44
152.59
139.96
137.75
132.62
129.87
129.55
128.87
128.72
128.28
121.65
119.96
117.89
116.86
113.95
113.67
55.14
54.99
165 160 155 150 145 140 135 130 125 120 115 110 105 100 95 90 85 80 75 70 65 60 55 50 45 40
f1 (ppm)

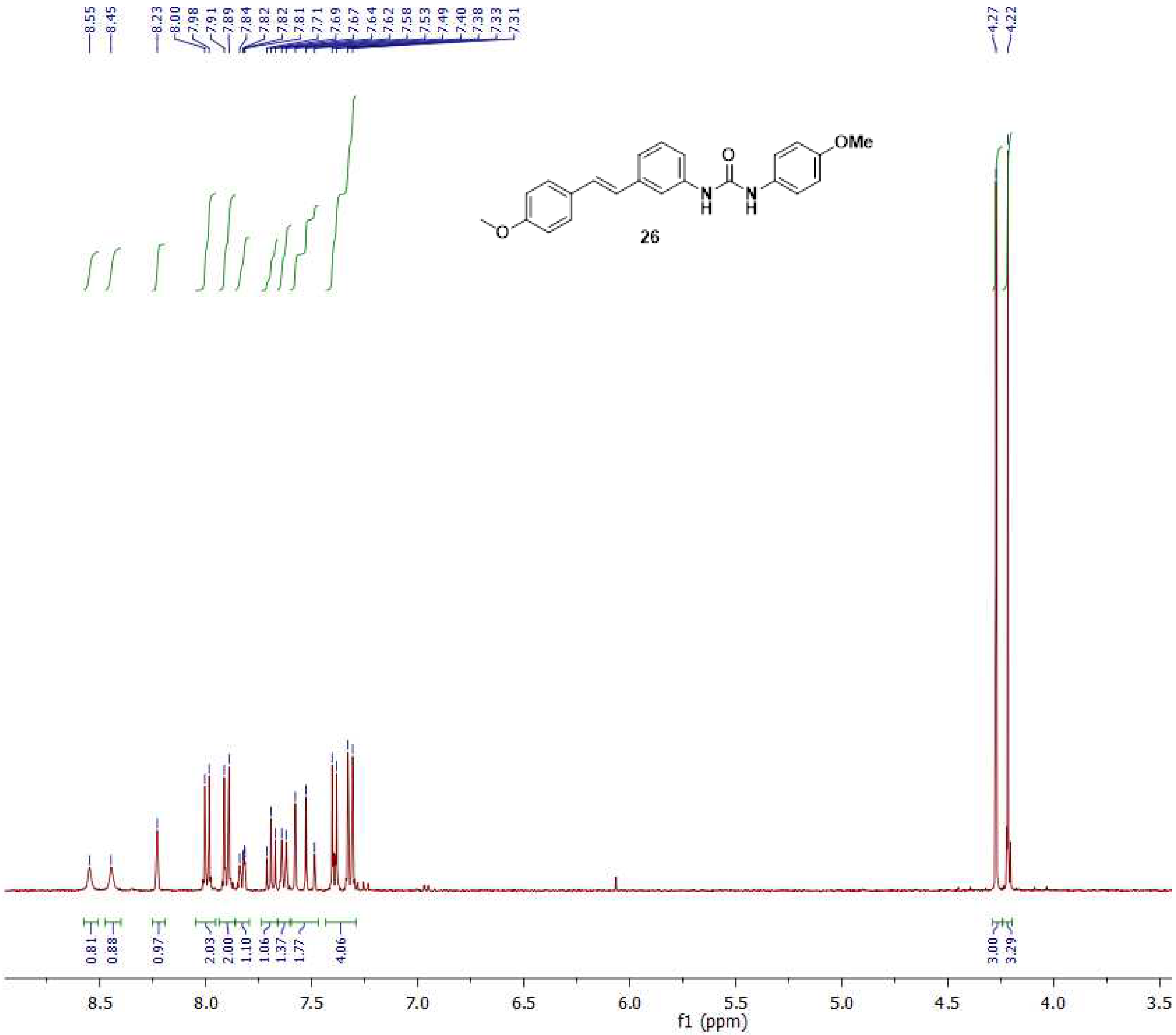
OMe
26
f1 (ppm)

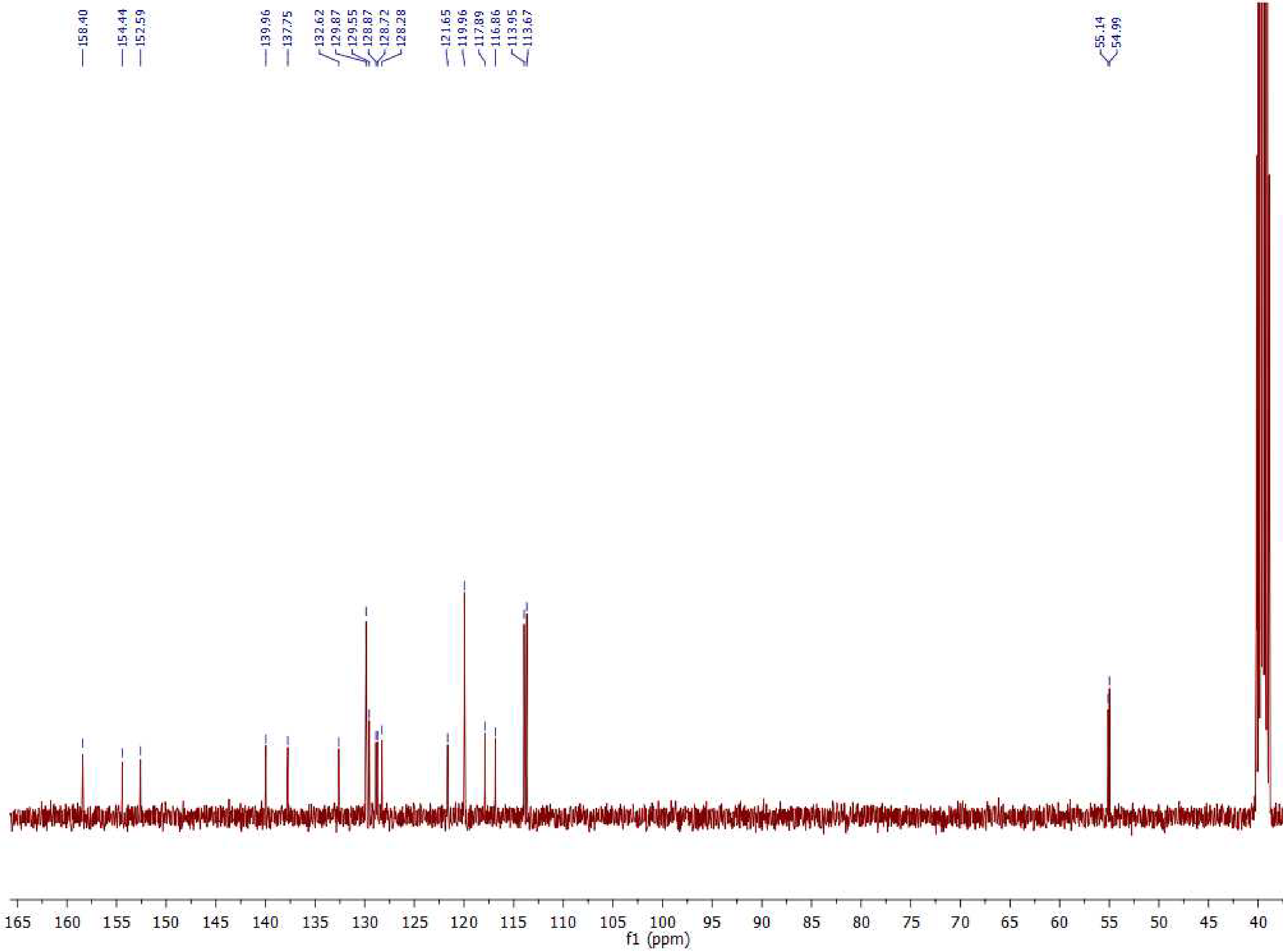
158.40
154.44
152.59
139.96
137.75
132.62
129.87
129.55
128.87
128.72
128.28
121.65
119.96
117.89
116.86
113.95
113.67
55.14
54.99
165 160 155 150 145 140 135 130 125 120 115 110 105 100 95 90 85 80 75 70 65 60 55 50 45 40
f1 (ppm)

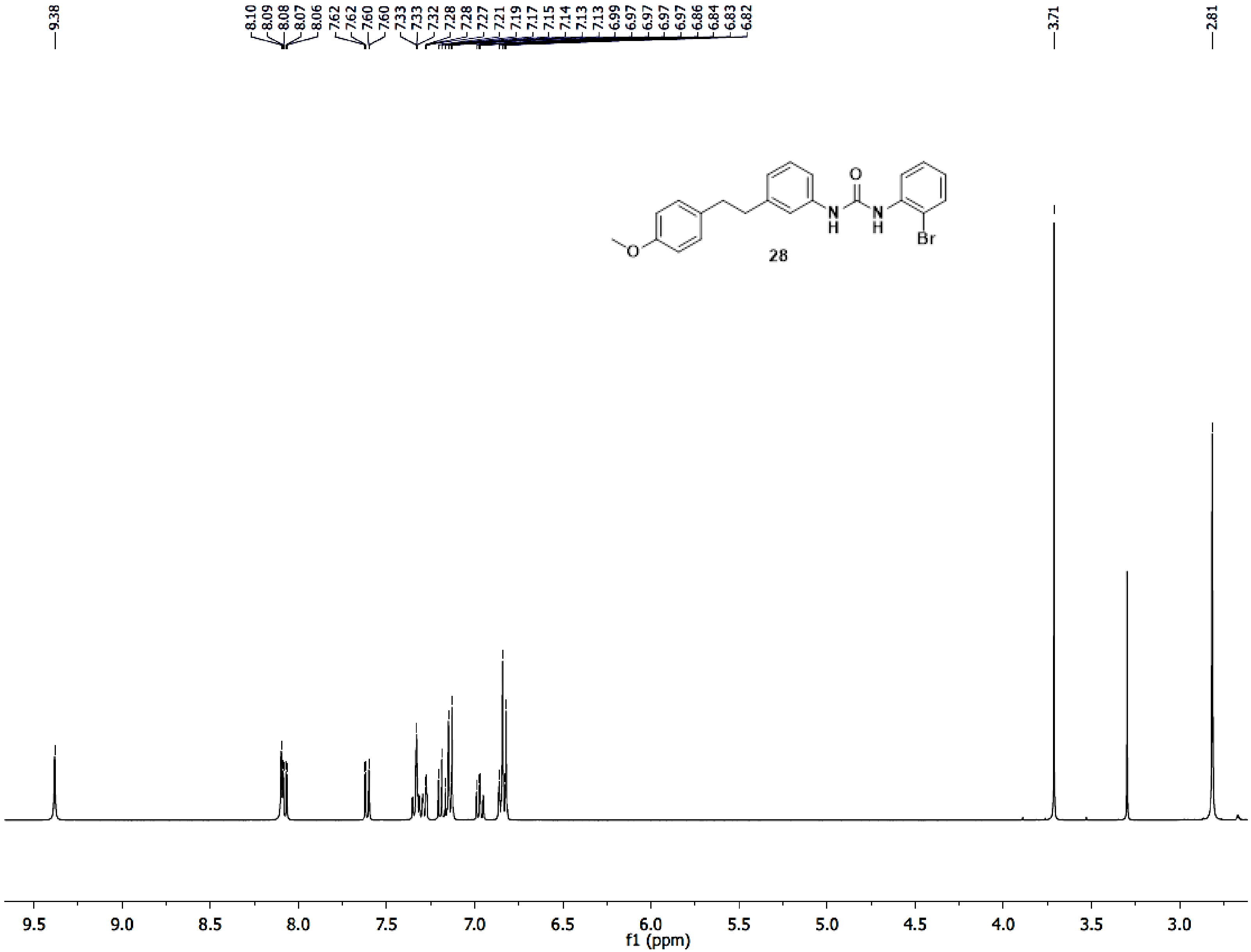
9.38
8.10
8.09
8.08
8.07
8.06
7.62
7.62
7.60
7.60
7.33
7.33
7.32
7.28
7.28
7.27
7.21
7.19
7.17
7.15
7.14
7.13
7.13
6.99
6.97
6.97
6.97
6.86
6.84
6.83
6.82
3.71
2.81
O
N
H
N
H
Br
O
28
9.5
9.0
8.5
8.0
7.5
7.0
6.5
6.0
5.5
5.0
4.5
4.0
3.5
3.0
f1 (ppm)

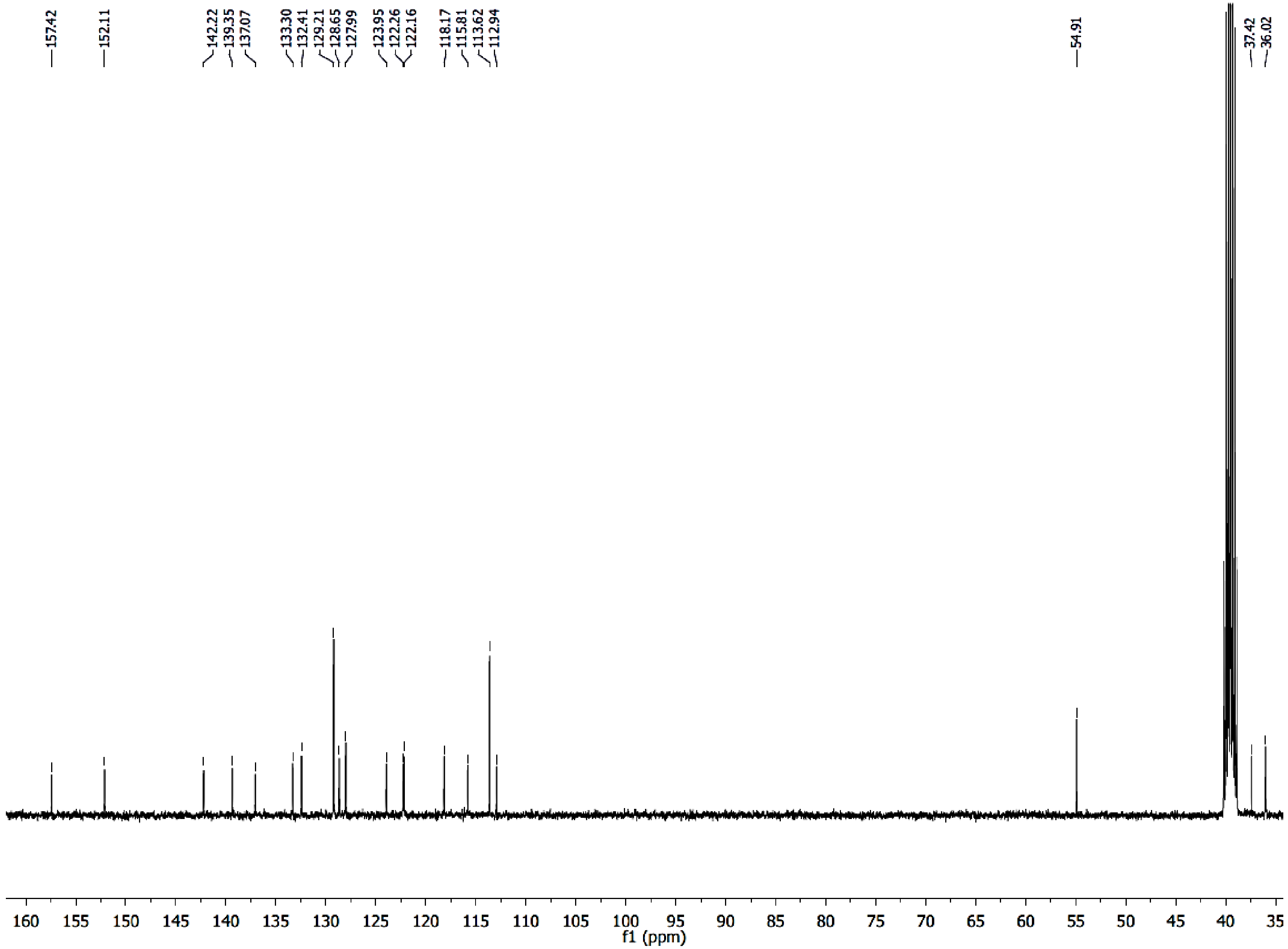
157.42
152.11
142.22
139.35
137.07
133.30
132.41
129.21
128.65
127.99
123.95
122.26
122.16
118.17
115.81
113.62
112.94
54.91
37.42
36.02
160 155 150 145 140 135 130 125 120 115 110 105 100 95 90 85 80 75 70 65 60 55 50 45 40 35
f1 (ppm)

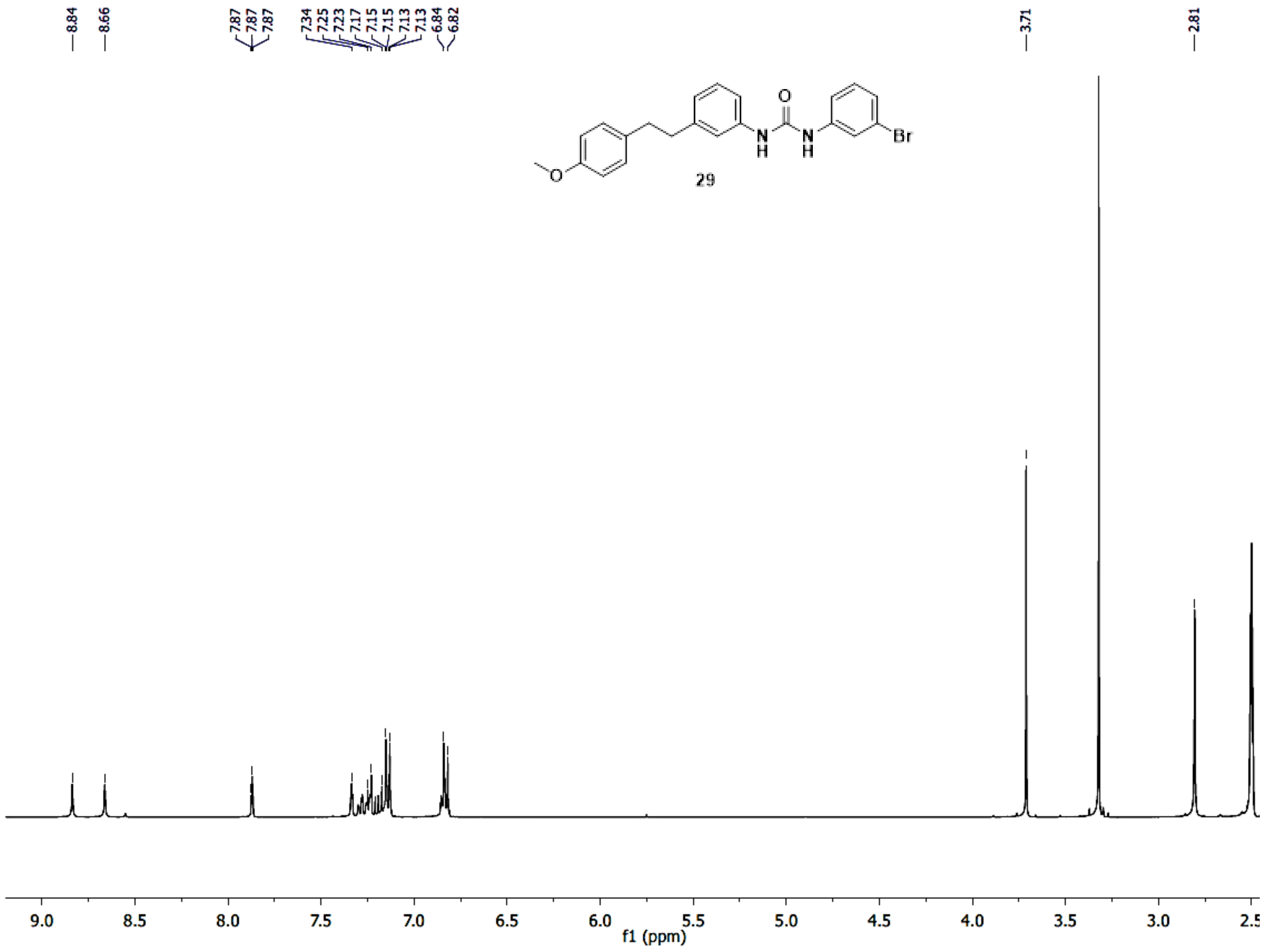
8.84
8.66
7.87
7.87
7.87
7.34
7.25
7.23
7.17
7.15
7.15
7.13
7.13
6.84
6.82
3.71
2.81
O
N
H
N
H
Br
29
9.0
8.5
8.0
7.5
7.0
6.5
6.0
5.5
5.0
4.5
4.0
3.5
3.0
2.5
f1 (ppm)

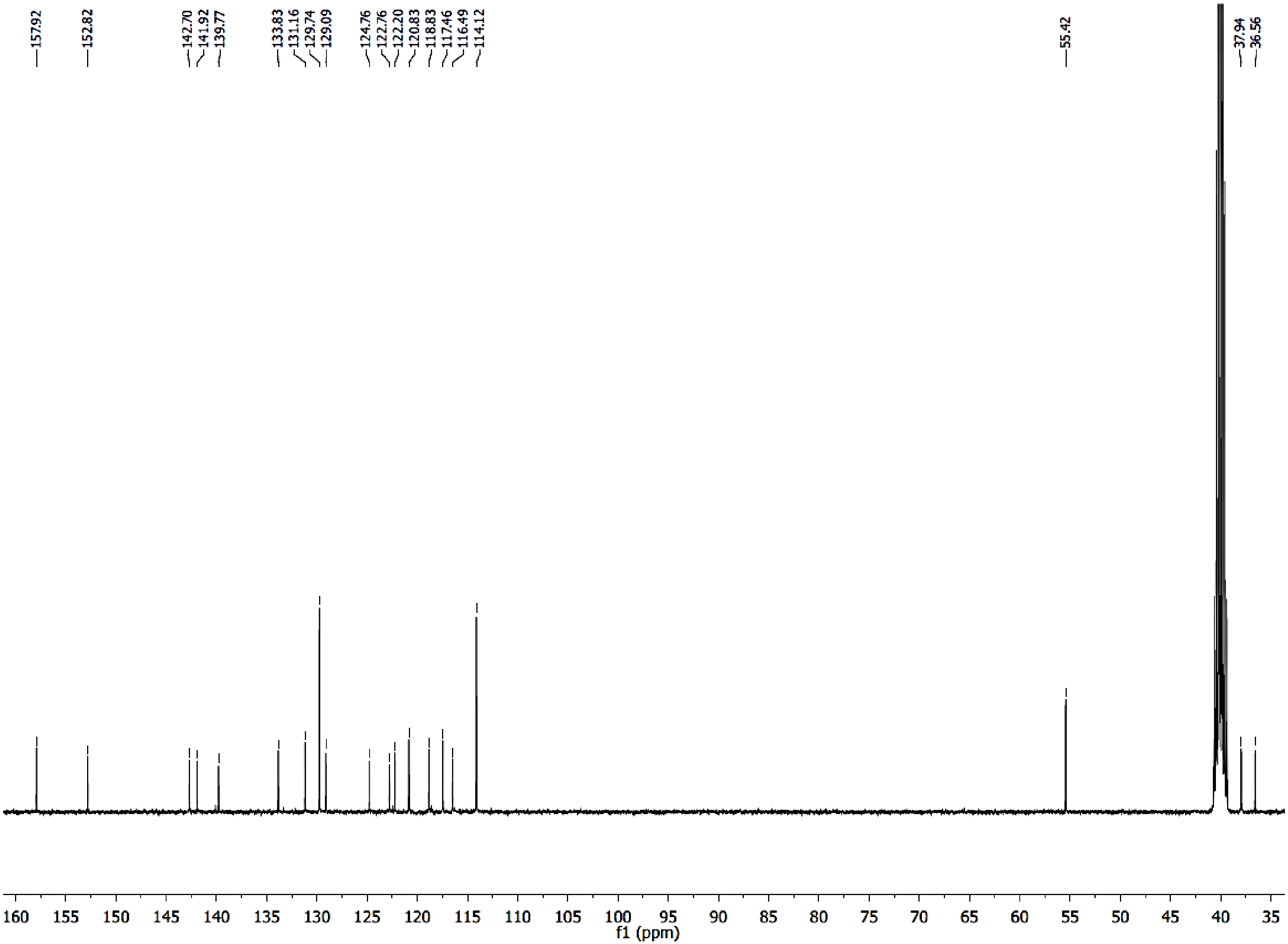
157.92
152.82
142.70
141.92
139.77
133.83
131.16
129.74
129.09
124.76
122.76
122.20
120.83
118.83
117.46
116.49
114.12
55.42
37.94
36.56
160 155 150 145 140 135 130 125 120 115 110 105 100 95 90 85 80 75 70 65 60 55 50 45 40 35
f1 (ppm)

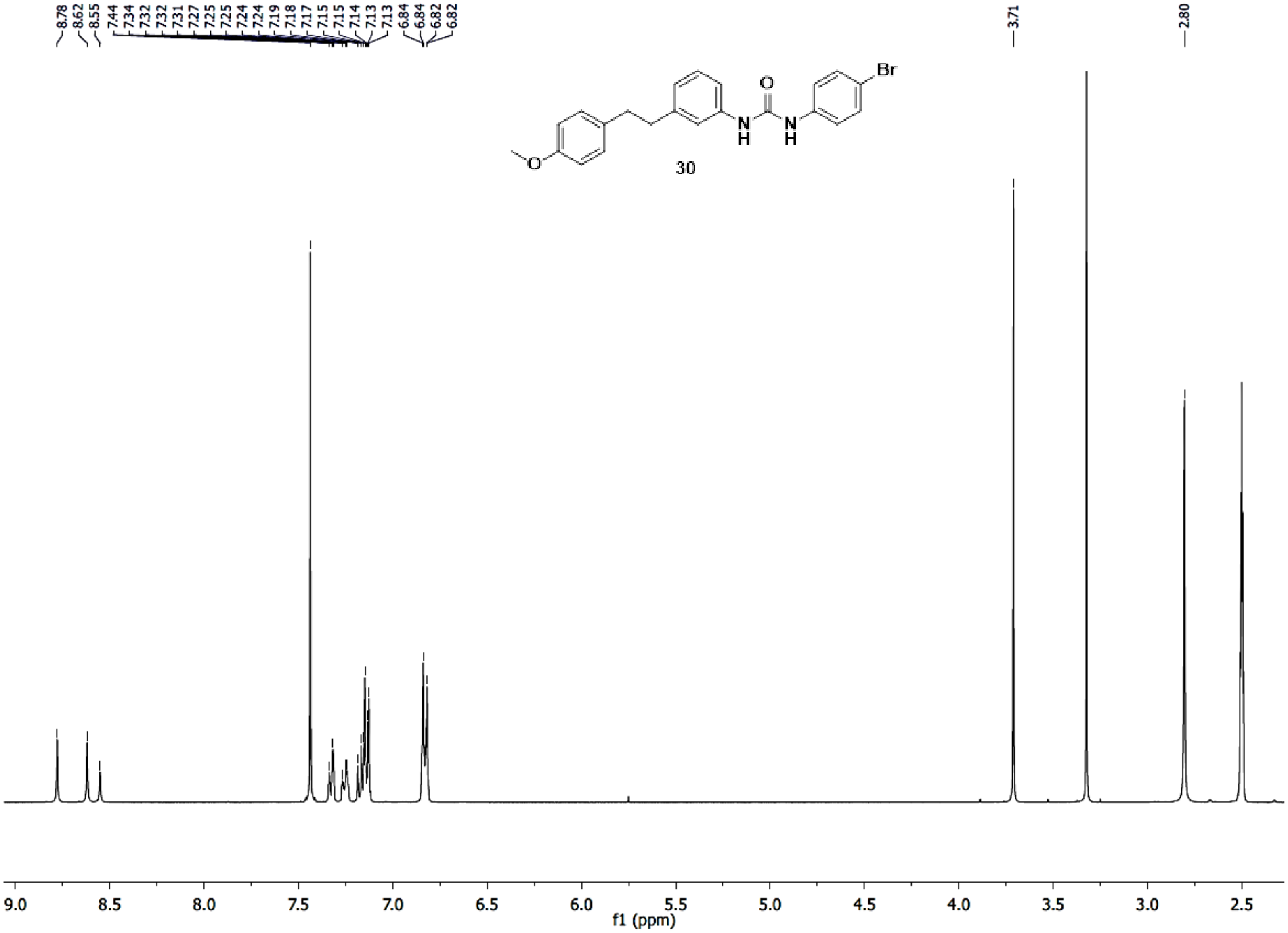
8.78
8.62
8.55
7.44
7.34
7.32
7.32
7.31
7.27
7.25
7.25
7.24
7.24
7.19
7.18
7.17
7.15
7.15
7.14
7.13
7.13
6.84
6.84
6.82
6.82
3.71
2.80
Br
O
N
H
N
H
O
30
9.0
8.5
8.0
7.5
7.0
6.5
6.0
5.5
5.0
4.5
4.0
3.5
3.0
2.5
f1 (ppm)

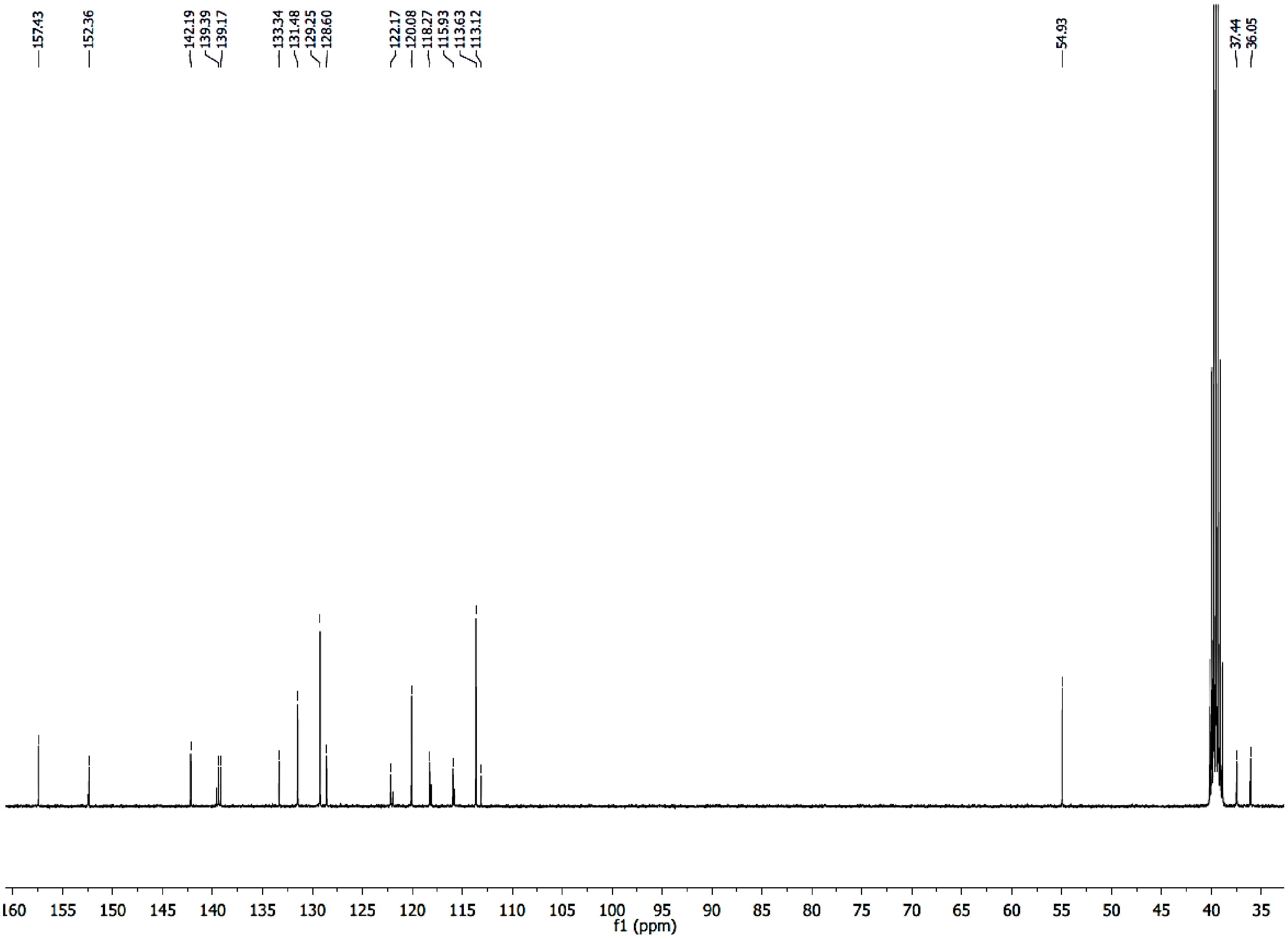
157.43
152.36
142.19
139.39
139.17
133.34
131.48
129.25
128.60
122.17
120.08
118.27
115.93
113.63
113.12
54.93
37.44
36.05
160 155 150 145 140 135 130 125 120 115 110 105 100 95 90 85 80 75 70 65 60 55 50 45 40 35
f1 (ppm)

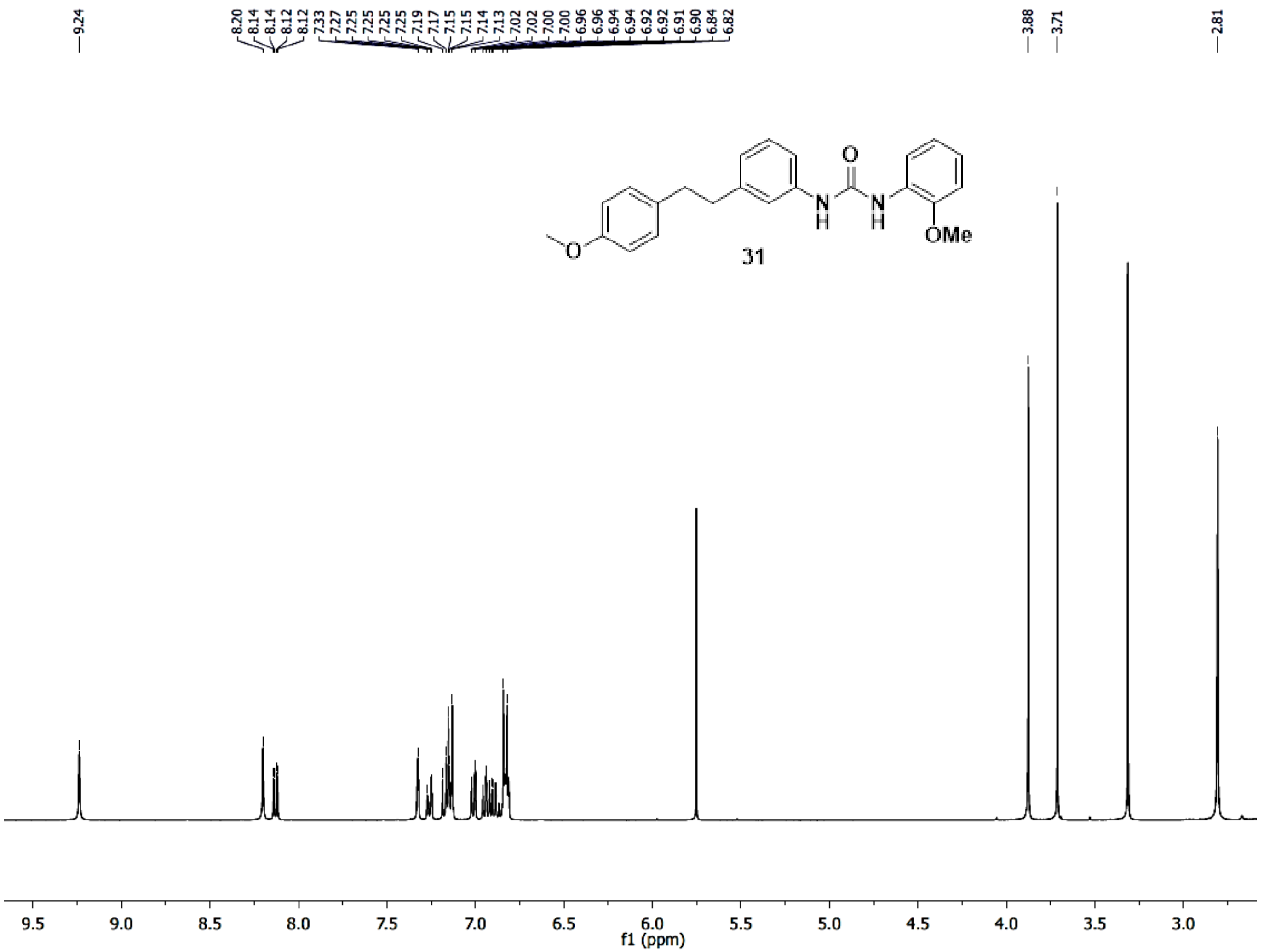

9.24
8.20
8.14
8.14
8.12
8.12
7.33
7.27
7.25
7.25
7.25
7.25
7.19
7.17
7.15
7.15
7.14
7.13
7.02
7.02
7.00
7.00
6.96
6.96
6.94
6.94
6.92
6.92
6.91
6.90
6.84
6.82
3.88
3.71
2.81
O
N
H
N
H
OMe
O
31
9.5
9.0
8.5
8.0
7.5
7.0
6.5
6.0
5.5
5.0
4.5
4.0
3.5
3.0
f1 (ppm)

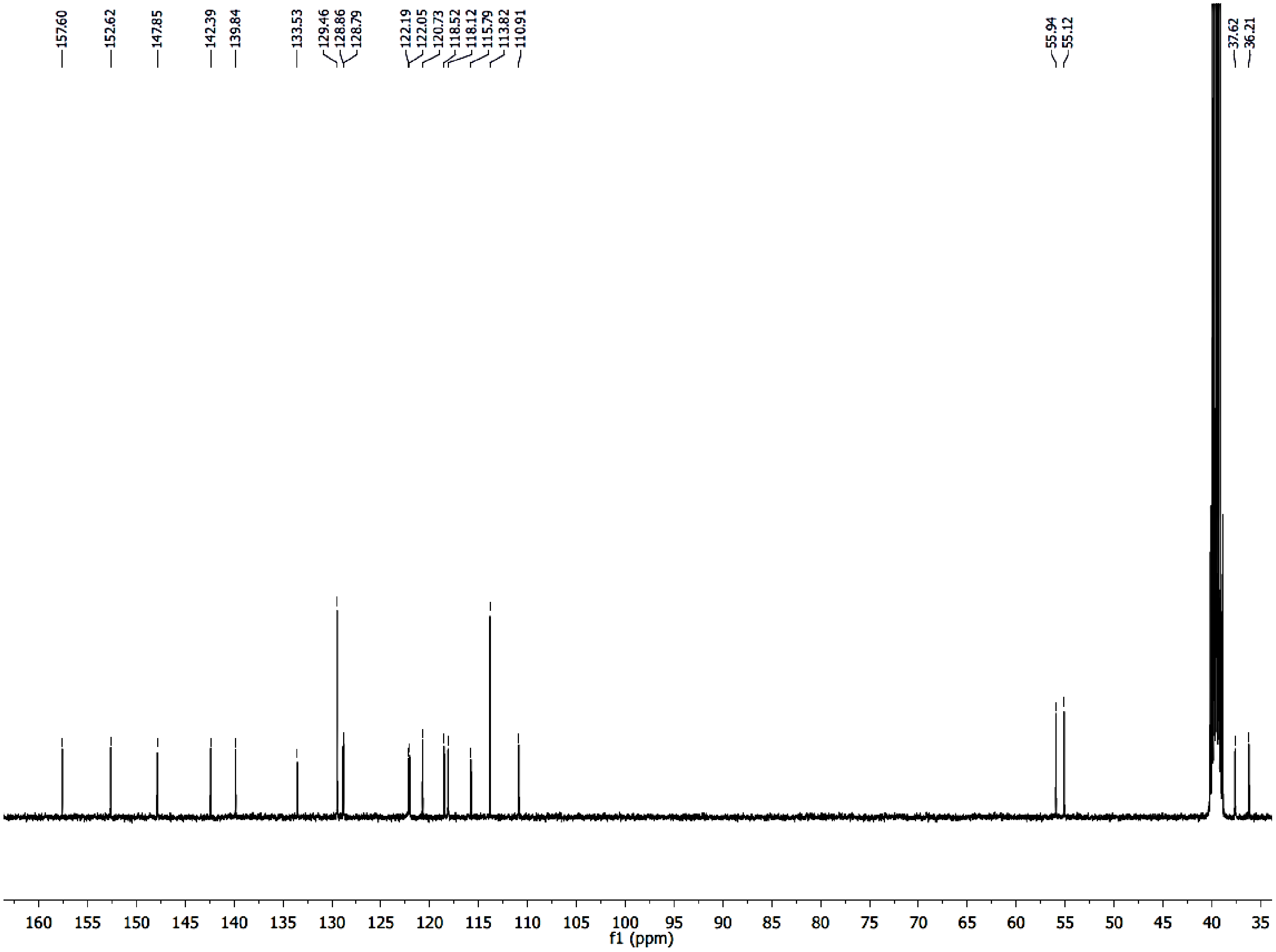
157.60
152.62
147.85
142.39
139.84
133.53
129.46
128.86
128.79
122.19
122.05
120.73
118.52
118.12
115.79
113.82
110.91
55.94
55.12
37.62
36.21
160 155 150 145 140 135 130 125 120 115 110 105 100 95 90 85 80 75 70 65 60 55 50 45 40 35
f1 (ppm)

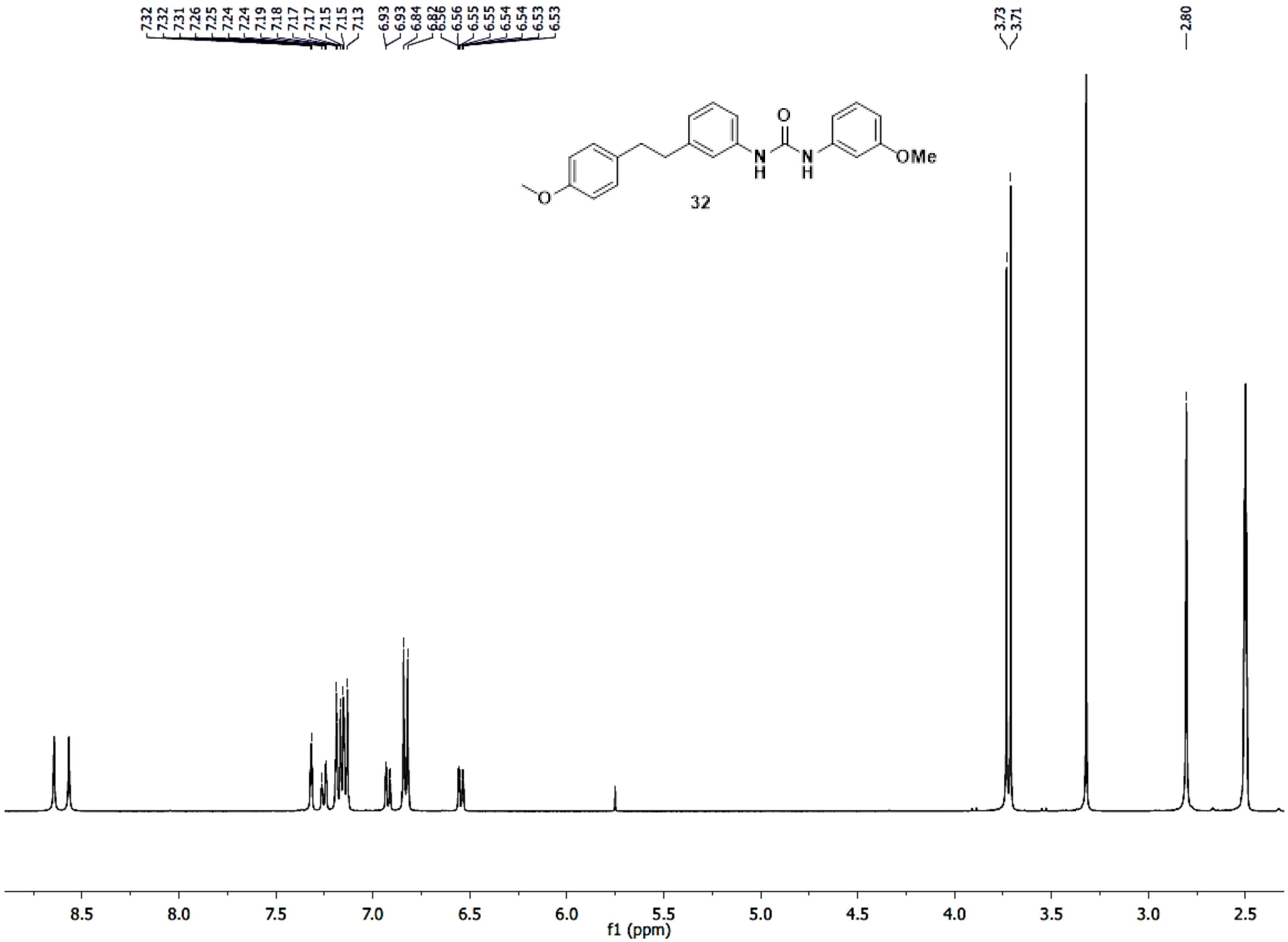
7.32
7.32
7.31
7.26
7.25
7.24
7.24
7.19
7.18
7.17
7.17
7.15
7.15
7.13
6.93
6.93
6.84
6.82
6.56
6.56
6.55
6.55
6.54
6.53
6.53
3.73
3.71
2.80
OMe
32
8.5
8.0
7.5
7.0
6.5
6.0
5.5
5.0
4.5
4.0
3.5
3.0
2.5
f1 (ppm)

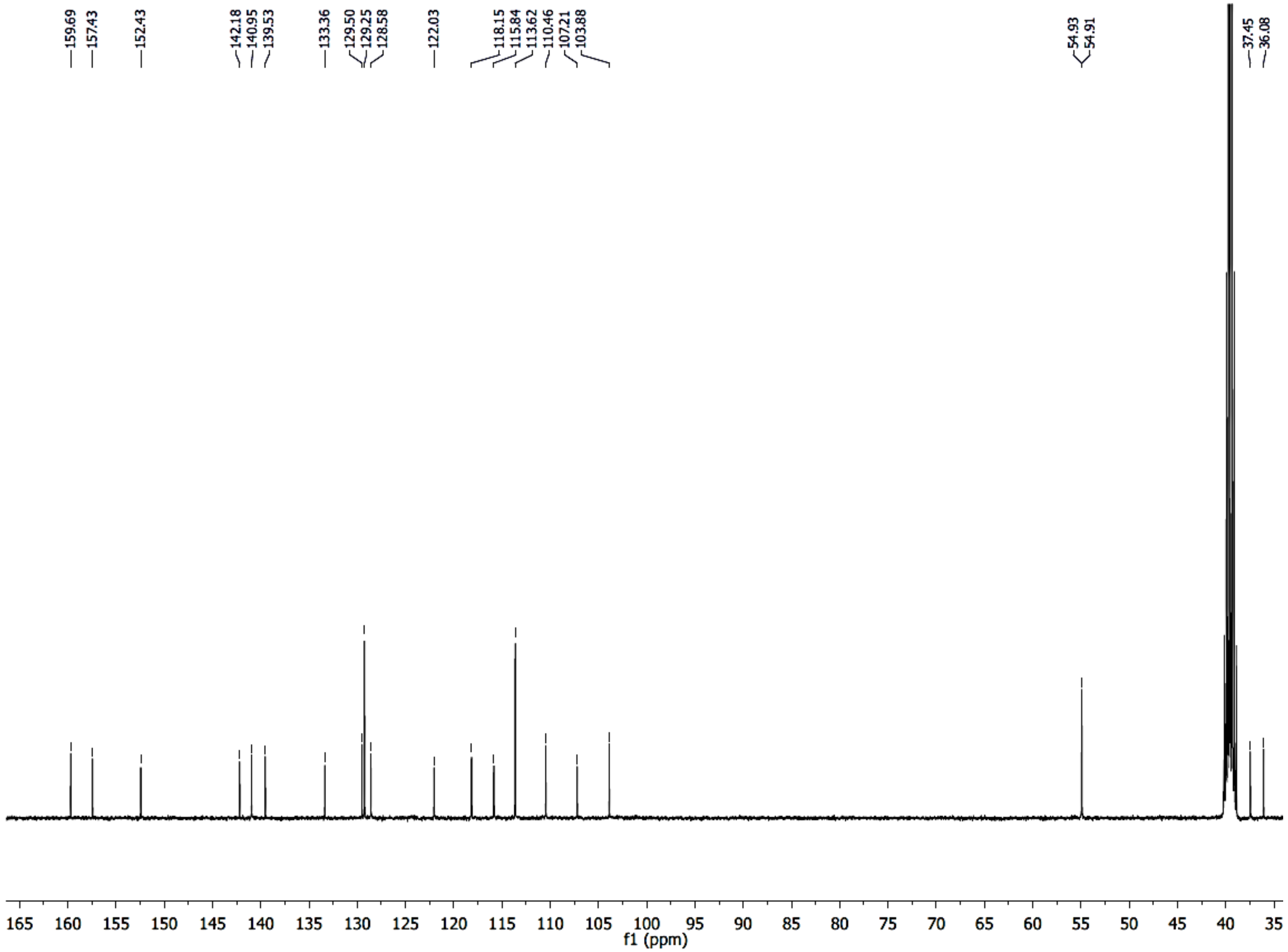
159.69
157.43
152.43
142.18
140.95
139.53
133.36
129.50
129.25
128.58
122.03
118.15
115.84
113.62
110.46
107.21
103.88
54.93
54.91
37.45
36.08
165 160 155 150 145 140 135 130 125 120 115 110 105 100 95 90 85 80 75 70 65 60 55 50 45 40 35
f1 (ppm)
